# The inotropic agent digitoxin strengthens desmosomal adhesion in cardiac myocytes in an ERK1/2-dependent manner

**DOI:** 10.1007/s00395-020-0805-3

**Published:** 2020-06-17

**Authors:** Camilla Schinner, Silvana Olivares-Florez, Angela Schlipp, Sebastian Trenz, Manouk Feinendegen, Heinrich Flaswinkel, Ellen Kempf, Desalegn Tadesse Egu, Sunil Yeruva, Jens Waschke

**Affiliations:** 1 Faculty of Medicine, Ludwig-Maximilians-Universität (LMU) Munich, Pettenkoferstraße 11, 80336 Munich, Germany; 2 Department of Biomedicine, University of Basel, Basel, Switzerland; 3 Department of Biology II, Ludwig-Maximilians-Universität (LMU) Munich, Munich, Germany

**Keywords:** Cardiomyopathies, Intercellular adhesion, Desmosome, Intercalated disc, Cardiac glycoside

## Abstract

Desmosomal proteins are components of the intercalated disc and mediate cardiac myocyte adhesion. Enhancement of cardiac myocyte cohesion, referred to as “positive adhesiotropy”, was demonstrated to be a function of sympathetic signaling and to be relevant for a sufficient inotropic response. We used the inotropic agent digitoxin to investigate the link between inotropy and adhesiotropy. In contrast to wild-type hearts, digitoxin failed to enhance pulse pressure in perfused mice hearts lacking the desmosomal protein plakoglobin which was paralleled with abrogation of plaque thickening indicating that positive inotropic response requires intact desmosomal adhesion. Atomic force microscopy revealed that digitoxin increased the binding force of the adhesion molecule desmoglein-2 at cell–cell contact areas. This was paralleled by enhanced cardiac myocyte cohesion in both HL-1 cardiac myocytes and murine cardiac slices as determined by dissociation assays as well as by accumulation of desmosomal proteins at cell–cell contact areas. However, total protein levels or cytoskeletal anchorage were not affected. siRNA-mediated depletion of desmosomal proteins abrogated increase of cell cohesion demonstrating that intact desmosomal adhesion is required for positive adhesiotropy. Mechanistically, digitoxin caused activation of ERK1/2. In line with this, inhibition of ERK1/2 signaling abrogated the effects of digitoxin on cell–cell adhesion and desmosomal reorganization. These results show that the positive inotropic agent digitoxin enhances cardiac myocyte cohesion with reorganization of desmosomal proteins in an ERK1/2-dependent manner. Desmosomal adhesion seems to be important for a sufficient positive inotropic response of digitoxin treatment, which can be of medical relevance for the treatment of heart failure.

**Electronic supplementary material:**

The online version of this article (10.1007/s00395-020-0805-3) contains supplementary material, which is available to authorized users.

## Introduction

For a coordinated contraction and structural integrity of the myocardium, mechanical and electrical coupling of the cardiac myocytes via the intercalated disc (ICD) is essential. In the heart, a mixed type of adhering junctions composed of desmosomes and adherens junctions known as area composita is present [10], whereas electrical coupling is mediated by gap junctions mainly consisting of connexin-43 (CX43). Furthermore, sodium channels with subunits Nav1.5 and β1 are present at the ICD and are involved in the conduction of electrical signals [15, 44, 45]. There is an increasing consensus in the literature that the single components of the ICD can not only be structurally intermingled but also act as functional unit with dependency on each other [6, 17, 34, 45]. In cardiac myocytes, desmosomes consist of the desmosomal cadherins desmoglein-2 (DSG2) and desmocollin-2, which extracellularly interact to form the intercellular adhesive interface and are intracellularly linked to desmin intermediate filament system via the adaptor proteins plakoglobin (PG), plakophillin-2 (PKP2) and desmoplakin (DP) [7]. In adherens junctions, the transmembrane adhesion molecule N-cadherin (NCAD) is coupled to the actin filament system [1]. The relevance especially of desmosomes is highlighted by the disease arrhythmogenic cardiomyopathy (AC), which is caused by mutations in genes encoding for desmosomal proteins [4]. AC becomes evident in patients by ventricular arrhythmia, which may cause sudden death with increased prevalence in young athletes. It is characterized by progressive loss of cardiac myocytes and fibrotic tissue replacement [41]. Physical exercise, which is paralleled with elevated adrenergic signaling, seems to be an important factor in the progression of the disease as it may aggravate mechanical uncoupling of cardiac myocytes and triggers malignant ventricular arrhythmias [4].

Recent data of our group showed that adrenergic stimulation with increased cAMP levels induces an elevation of cell–cell adhesion in cardiac myocytes with remodeling of desmosomal proteins at the ICDs—an effect referred to as “positive adhesiotropy” [35, 53]. This effect was shown to be dependent on PKA-mediated phosphorylation of PG at Serine 665. Compared to controls, hearts of a PG-deficient AC mouse model were incapable of increasing intercellular adhesion in response to adrenergic stimulation. Furthermore, these hearts failed to increase pulse pressure—a correlate for positive inotropy, which is one of the main functions of adrenergic stimulation to adapt heart function to increased physical load.

As these data suggest a dependency of increase in contractility (positive inotropy) on strengthened cell–cell adhesion (positive adhesiotropy), we here aim to investigate the interplay of these adrenergic effects in cardiac myocytes in more detail. For this purpose, we applied the cardiac glycoside digitoxin as a positive inotropic agent. Digitoxin has been in clinical use for the treatment of congestive heart failure and cardiac arrhythmias [28]. As mode of action, digitoxin is assumed to inhibit the Na^+^/K^+^-ATPase in cardiac myocytes leading to an accumulation of Ca^2+^ with elevated contractility and higher contraction forces [11, 27, 32, 38, 51]. In addition, there is also evidence that digitoxin can increase contractility e.g. via binding to ryanodine receptors [11, 51].

In this study, we demonstrate that the positive inotropic agent digitoxin strengthens adhesion between adjacent cardiac myocytes in a desmosome-dependent manner with recruitment of DSG2, DP, and PG at cell–cell contacts paralleled with thickening of the ICD plaque. This process is shown to be dependent on activation of ERK1/2, while phosphorylation of PG as described for adrenergic stimulation was not involved. On the other hand, adrenergic stimulation was shown to phosphorylate ERK1/2 in addition to phosphorylation of PG, whereas no additive effect to digitoxin was detectable. Murine hearts lacking PG failed to adapt pulse pressure in response to digitoxin treatment and thickening of the ICD plaque was abrogated. These data show that an inotropic agent strengthened cohesion of the ICD and indicate that intact desmosomal cohesion parallel to elevated contractility is important for sufficient heart function. These findings might be important for treatment strategies in heart failure and AC.

## Methods

### Mediators

Digitoxin (Sigma-Aldrich,  St. Louis, Missouri, USA, #D5878) was dissolved in 50% ethanol/50% polyethylene glycol (Sigma-Aldrich) and applied for 60 min at concentrations as indicated. The combination of the adenylyl cyclase activator forskolin and phosphodiesterase-4 inhibitor rolipram (*F*/*R*, Sigma-Aldrich, #F6886/#R6520) was dissolved in dimethyl sulfoxide (DMSO) and applied for 60 min at concentrations of 5 μM or 10 μM, respectively. Combination of *F*/*R* 5 μM/10 μM and digitoxin 1 μM were applied simultaneously for 60 min. The MEK1/2 inhibitor UO 126 (Sigma-Aldrich, #19-147) was dissolved in DMSO and pre-incubated for 60 min before incubation with digitoxin at concentrations as indicated. For control conditions, corresponding solvents were applied in the same amount and time as the indicated mediators.

### Cell culture

The murine cardiac myocyte cell line HL-1 was provided by William Claycomb (LSU Health Sciences Center, New Orleans, USA) and cultured according to his instructions in Claycomb medium supplemented with 100 µM norepinephrine, 10% fetal bovine serum, 100 µg/ml penicillin/streptomycin and 2 mM l-glutamine at 37 °C, 5% CO_2_ and 100% humidity. Cells were seeded on glass cover slips for immunostaining or AFM experiments or on 24-well plates for Western blot analysis or Triton-X-extraction assay pre-coated with 25 µg/ml bovine fibronectin in 0.02% gelatin solution (Sigma-Aldrich). After seeding for experiments, cells were incubated in Claycomb medium additionally supplemented with 1.8 mM Ca^2+^ to ensure adequate cadherin cohesion and without norepinephrine to reduce basal adrenergic stimulation.

### Generation of monoclonal antibody against p-PG-S665

A monoclonal antibody against p-PG-S665 was generated as described previously [9, 53]. In short, a peptide comprising amino acids 656-DYRKRVpSVELTNS-671 from human PG was synthesized and coupled to OVA (Peps4LS, Heidelberg, Germany). C57BL/6 were immunized subcutaneously and intraperitoneally with a mixture of 50 µg peptide-OVA, 5 nmol CPG oligonucleotide (Tib Molbiol, Berlin), 100 µl phosphate buffered saline (PBS) and 100 µl incomplete Freund's adjuvant. A boost without adjuvant was given six weeks after the primary injection. Fusion was performed using standard procedures. Supernatants were tested in a differential ELISA with the phosphorylated peptide or the non-phosphorylated peptide, both coupled to bovine serum albumin. Monoclonal antibodies that reacted specifically with the phospho-peptide were further analyzed in Western blot. Tissue culture supernatant of clone 1B8 (mouse IgG1, kappa) was used in this study.

### Dissociation assay in vitro

Confluent HL-1 cells were treated as indicated and incubated with dissociation buffer (liberase DH 0.065 U/ml, Roche, Basel, Switzerland; dispase II 2.5 U/ml, Sigma-Aldrich) at 37 °C until detachment of intact cell monolayers from the well bottom. After replacement of the dissociation buffer by Hank’s balanced salt solution (HBSS), the monolayers were mechanically stressed by horizontal rotation at 1250 rpm for 5 min. The resulting monolayer fragmentation was determined using a binocular stereomicroscope (Leica microsystems, Wetzlar, Germany). To test for Ca^2+^ insensitivity of cell cohesion, HL-1 monolayer were detached from well bottom by dissociation buffer and pre-incubated with indicated mediators for 60 min in Claycomb medium with 1.8 mM Ca^2+^ added. After switching to medium containing 5 mM of the Ca^2+^ chelator ethylene glycol-bis(2-aminoethylether)-*N*,*N*,*N*′,*N*′-tetraacetic acid (EGTA) and the corresponding mediators, floating monolayers were incubated further for 90 min. Subsequently, monolayers were subjected to mechanical shear stress as described above. Per independent experiment, duplicates were performed per condition with their mean value taken for statistical analysis.

### Immunostaining

Immunostainings were performed using standard procedures. Details and antibodies are provided in Supplementary data—Supplementary methods. Samples were imaged and analyzed with the Leica SP5 confocal microscope equipped with a 63 × oil objective using LAS-AF software (all Leica microsystems). For HL-1 cells, a maximum intensity projection of 10 consecutive images with 0.3 µm step height is shown. For analysis of signal intensity at the ICDs, area masks of the ICDs were created in the DP images and applied to the corresponding DSG2 and DP images to measure mean signal intensity in these areas. The corresponding mean signal background was subtracted from all values.

### Western blot analysis

Western blot analysis was performed using standard procedures. Details and applied antibodies are provided in Supplementary data—Supplementary methods. Densitometric band analysis was performed with Image Studio Lite Ver. 5.2 (LI-COR Biosciences, Bad Homburg, Germany).

### Triton-X-extraction assay

HL-1 cell lysates were separated into cytoskeleton-anchored (Triton-insoluble, pellet) and non-cytoskeleton-anchored (Triton-soluble, supernatant) protein fractions using standard procedures [14]. Cells were treated as indicated and incubated with Triton buffer (0.5% Triton-X-100, 50 mM MES, 25 mM EGTA and 5 mM MgCl_2_) on ice for 10 min. To separate the fractions, cells were scraped and centrifuged at 14,000 *G* for 10 min at 4 °C. The resulting supernatant (non-cytoskeletal-bound faction) was collected and the pellet (cytoskeletal-bound fraction) was lysed in SDS-lysis buffer. Western blot analysis was performed as described above.

### Membrane isolation

Isolation of membrane fraction was performed according to [23]. Confluent HL-1 cells were treated as indicated, washed with ice-cold PBS and lysed in cell lysis buffer (20 mM Tris, pH 7.5, 5 mM EDTA, 2 mM EGTA, supplemented with a protease-inhibitor cocktail (Complete-O, Roche). After sonication, lysates were centrifuged at 20,000 *G* for 60 min. The resulting supernatant (cytosolic fraction) was collected and the pellet was lysed in membrane isolation buffer (1% Triton X-100, 20 mM Tris, pH 7.5, 5 mM EDTA, 2 mM EGTA, supplemented with a protease-inhibitor cocktail (Complete-O, Roche), incubated for 60 min on ice, and centrifuged at 20,000 *G* for 60 min. The resulting supernatant (membrane fraction) was collected. Both fractions were subjected to Western blot analysis as described above.

### FURA-2 Ca^2+^ measurement

HL-1 cardiac myocytes were loaded with 0.4% Fura-2-AM (Molecular Probes, Thermo Fisher) and 0.4% Pluronic F-127 (Life Technologies, Thermo Fisher) diluted in measurement buffer (140 mM NaCl, 3.6 mM KCl, 2.6 mM CaCl_2_, 0.5 mM NaH_2_PO_4_, 2 mM NaHCO_3_, HEPES and 5 mM d-glucose) by incubation for 20 min at 37 °C. After washing in measurement buffer and incubation for 15 min, analysis was started. The experiments were performed with an inverted microscope (Carl Zeiss Microscopy) equipped with a polychrome V light source (TILL Photonics, Martinsried, Germany) a CoolSNAP-Hq2 digital camera (Photometrics) and a FURA-2 filter set (AHF Analysetechnik, Tuebingen, Germany). Caffein (Sigma-Aldrich, C0750, solved in water) was applied at 10 µM as positive control.

### Phos-Tag

To determine the phosphorylation state of HL-1 cells, manganese(II)-Phos-Tag SDS-PAGE was performed according to manufacturer’s instructions (Wako Chemicals GmbH, Steinbach, Germany). Treated HL-1 cells were lysed in modified RIPA-buffer (10 mM Na_2_HPO_4_, 150 mM NaCl, 1% Triton-X-100, 0.25% SDS, 1% Na desoxycholate, pH 7.2) supplemented with leupeptin, aprotenin, pepsatin, phenylmethylsulfonyl fluoride, and phosphatase inhibitors (Roche), mixed with Laemmli buffer and loaded on a freshly prepared 6%-polyacrylamide gel containing either 0 mM as control or 30 mM Phos-Tag (ALL-107, Wako Chemicals) to separate phosphorylated proteins. Protein detection was performed as described above.

### siRNA-mediated knock-down

To specifically reduce protein levels, 60–70% confluent HL-1 cells were transfected with ONTARGET plus SMARTpool mouse Jup-siRNA (siPG), Dsg2-siRNA (siDSG2) or Dp-siRNA (siDP) in parallel with non-targeting siRNA (siNT) as control (all Dharmacon, Thermo Fisher Scientific, Layfatte, USA) using RNAiMAX (Thermo Fisher Scientific) according to manufacturer’s protocol. siRNA was incubated for 24 h and cells were grown to confluency within four days. Knockdown efficiency was confirmed by Western blot analysis for every experiment.

### Overexpression of PG constructs

Cloning of human wild-type PG tagged with GFP (PG-WT-GFP) and the corresponding phospho-deficient mutant PG-S665A-GFP were described previously [35]. Transient transfections with 1 µg of the above plasmids were performed 24 h post seeding in HL-1 cells using TurboFect transfection reagent (Thermo Fisher Scientific) according to manufacturer’s protocol. 72 h post-transfection, cells were treated with respective reagents and processed for immunostaining.

### Atomic force microscopy

Force spectroscopy on living HL-1 cardiac myocytes was performed as described previously [35]. Briefly, eukaryotically expressed DSG2-Fc protein was coupled to Si3N4 AFM cantilevers (MLCT probes, Bruker, Calle Tecate, CA, USA) in a concentration of 0.15 mg/ml via a bifunctional polyethylene glycol spacer (acetal-PEG-NHS, Gruber Lab, Institute of Biophysics, Linz, Austria). Experiments were performed with the pyramidal-shaped D-tip (nominal spring constant: 0.03 N/m) clamped into a Nanowizard III atomic force microscope (JPK instruments, Berlin, Germany) mounted on an optical fluorescence microscope (Axio Observer D1, Carl Zeiss) at 37 °C. SPM Control v.4 software (JPK instruments) was used for data acquisition. Force measurements were performed in a region of 5 μm × 1.25 μm spanning the cell–cell contact area of HL-1 cells with following settings: 64 × 16 pixel grid, setpoint 0.2 nN, extend speed 5 μm/s, extend delay 0.1 s. To determine the binding force between the tip-coupled DSG2 and the proteins on the cell surface, the functionalized tip was repeatedly approached to and retracted from the surface. If binding between the tip and surface occurred, the force necessary to rupture the binding (termed as “unbinding force”) was detected by determination of the cantilever’s deflection. The same cell–cell border was compared before and after addition of digitoxin.

### Mouse models

Animal handling and sacrifice was done with the approval of Ludwig-Maximilians-Universität Munich, Germany and of the European Commission according to the guidelines from Directive 2010/63/EU of the European Parliament on the protection of animals used for scientific purposes. All mice were obtained from the institutes’ animal facility and sacrificed by cervical dislocation. For experiments without genetically modified mice, adult 10–12-week-old C57BL/6 mice were used. PG-deficient mice (PG KO) were generated and characterized as described previously [35]. In brief, mice with loxP sites flanking exon 1 of the junctional plakoglobin gene (*Jup*) on both alleles (*Jup*^*tm1.1Glr*^/J mice, the Jackson Laboratory, Bar Harbor, USA) were bred with mice heterozygous for loxP sites in *Jup* with expression of the recombinase Cre under control of the cardiac specific alpha myosin heavy chain promotor (*Myh6*), which were generated by crossing with B6.FVB-Tg(Myh6-cre)2182Mds/J mice (The Jackson Laboratory).

### Dissociation assay in cardiac slices

Cardiac myocyte cohesion in murine ventricular tissue was determined as established previously [35]. 10–12-week-old C57BL/6 wild-type mice were obtained from the institutes’ animal facility and sacrificed by cervical dislocation. 200 μm thin murine cardiac slices were prepared as described above and transferred to cardiac slice medium (DMEM 1:1 F12 nutrient mixture, 20.4% knockout serum replacement, 1% minimum essential medium nonessential amino acids and 0.1% 2-mercaptoethanol, 2 mM l-glutamine, 10 U/L penicillin and 10 μg/ml streptomycin) (all purchased from Thermo Fisher Scientific) and incubated with indicated mediators at 37 °C, 5% CO_2_. To control variations because of different slice size or location in the ventricle, consecutive slices were used for control and treatment, respectively. After incubation in dissociation buffer, slices were subjected to similar mechanical stress by repeated pipetting with an electric pipette. After staining intact viable cardiac myocytes with thiazolyl blue tetrazolium bromide (Sigma-Aldrich), the number of dissociated cardiac myocytes was counted with an inverted microscope (Axio, Carl Zeiss, Oberkochen, Germany) and taken as indirect measurement for intercellular adhesion.

### Langendorff heart preparation

Ex vivo heart perfusion according to Langendorff was performed as described previously [36]. 12-week-old PG KO with their corresponding wild-type (WT) littermates were obtained from the institutes’ animal facility and sacrificed by cervical dislocation. Experiments were performed on a commercially available apparatus (AD Instruments, Spechbach, Germany) and recorded using Labchart7 software (AD Instruments). Digitoxin was applied at 100 nM or 1 µM for 15 min and 10 min respectively using a perfusion pump. Pulse pressure was measured via an aortic cannula. ECG was recorded with incision electrodes.

### Transmission electron microscopy and quantitative analysis

300 µm thick murine cardiac slices as described in the section “Dissociation assay in cardiac slices” were fixed after the respective treatments with 1% glutaraldehyde in PBS for 2 h at 4 °C. After washing with PBS for three times, tissue was post-fixed in 2% osmium tetroxide, dehydrated through an ascending ethanol series subsequently embedded in EPON 812 (Serva Electrophoresis GmBH, Heidelberg, Germany) and cured at 80 °C for 24 h. After trimming, ultrathin sections (60 nm) were cut with a diamond knife (DiATOME Electron Microscopy Sciences, Hatfield, USA) and placed on copper/rhodium grids (150 mesh, Plano GmbH, Wetzlar, Germany). Contrasting was performed with a saturated uranyl acetate solution and lead(II)citrate according to standard protocols [29, 50]. Images were acquired using a Libra 120 transmission electron microscope (Carl Zeiss) equipped with a SSCCD camera system (TRS, Olympus, Tokyo, Japan).

For quantitative analysis, 25 ICDs per condition and mouse were analyzed by a blindfolded observer using ImageJ software (NIH, USA). Plaque thickness was determined by measuring from the lateral end of one Plaque to the lateral end of the plaque of the adjacent cell and subtracting the measured intercellular space. Measurement was done three times per ICD and averaged to level out differences within one ICD. ICD length was measured by following the convolution of each ICD.

### Image analysis and processing

ImageJ (NIH) was used for image analysis and Photoshop CS5 (Adobe, San José, USA) for image processing and compilation. To create and analyze graphs and plots, Origin 9.1 (Originlab, Northampton, MA, USA), Excel (Microsoft, Redmond, WA) and GraphPad prism 5 (GraphPad Software, La Jolla, USA) were applied. Western blot bands were analyzed using Image Studio Ver 5.2 (LI-COR Biosciences). To analyze AFM force distance curves and process AFM images, JPK data processing software (JPK instruments) was used. Labchart7 software (AD Instruments) was applied to analyze heart rate and pulse pressure of Langendorff heart preparations.

### Statistics

Statistical analysis was performed using GraphPad Prism 5 (GraphPad Software, La Jolla, USA) as described in the corresponding figure legend. Normal distribution was tested for every data set by Shapiro–Wilk test. If necessary, log10 transformation was performed for normal distribution. Statistical significance was assumed at *P* < 0.05. Data are expressed as mean ± standard deviation (SD).

## Results

### The positive inotropic effect of digitoxin is dependent on PG

**Fig. 1:**
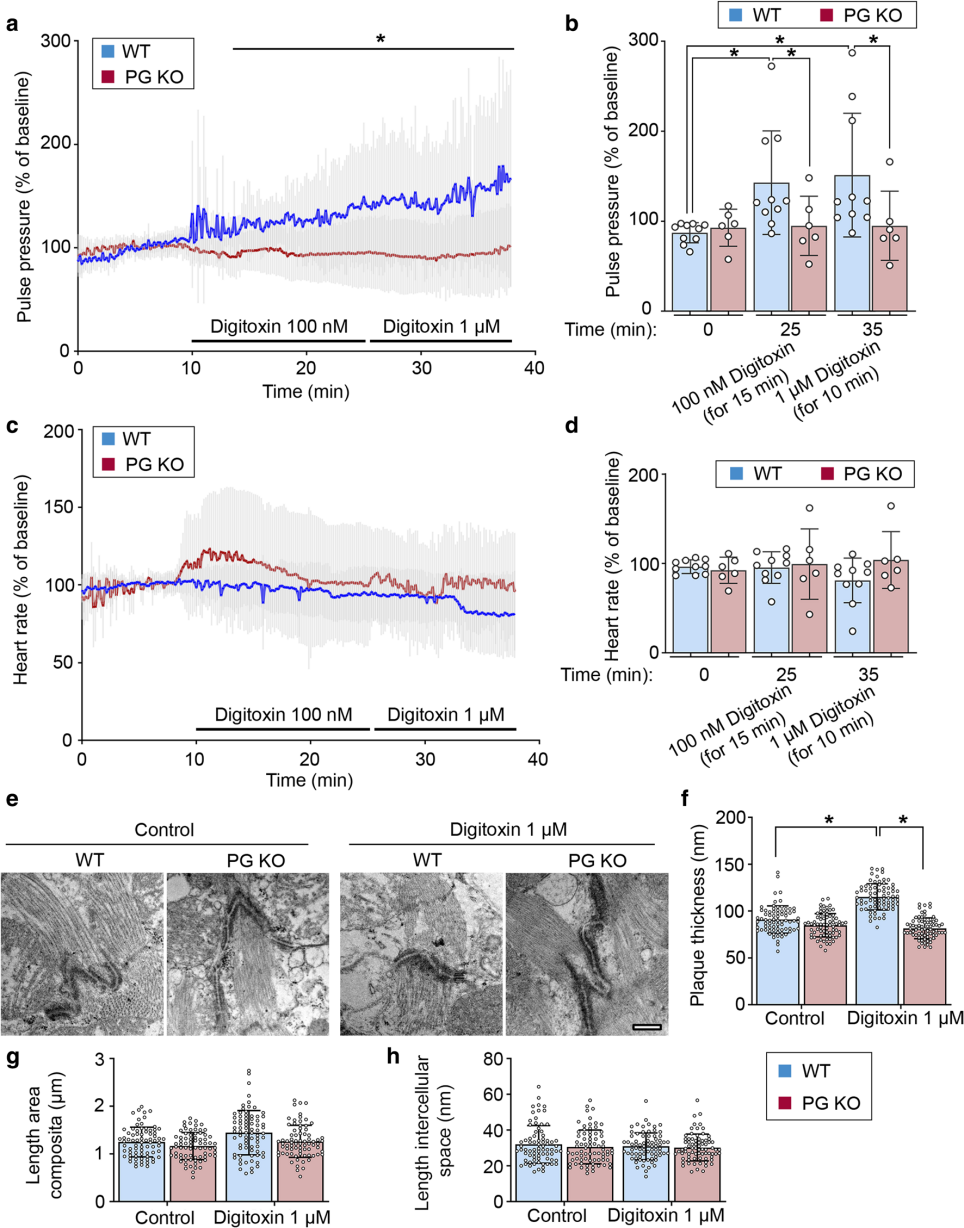
Positive inotropy induced by digitoxin is dependent on plakoglobin (PG) in ex vivo-perfused hearts. Mean pulse pressure (**a**) and heart rate (**c**) curve of hearts of 12-week-old WT or PG KO mice perfused with indicated digitoxin concentrations in an ex vivo Langendorff setting. Lines indicate mean values ± SD. Two-tailed, unpaired Student’s *t *test, *P < 0.05 vs. WT. **b**, **d** Represent corresponding values of *N* = 10 mouse hearts for WT and 6 mouse hearts for PG KO at indicated time points. Every dot corresponds to one individual heart, mean ± SD. Two-way ANOVA with Tukey’s post-hoc test. **e** Representative transmission electron microscopy images from cardiac slices derived from hearts of 12-week-old WT or PG KO mice and treated with digitoxin 1 µM for 60 min. Scale bar: 375 nm. *N* = 3 mice per condition. Analysis of junctional plaque thickness (**f**), length of area composita junctions (**g**), and length of intercellular space (**h**) corresponding to **e**. Every dot corresponds to one ICD, mean ± SD. Two-way ANOVA with Tukey’s post-hoc test

To first of all investigate if desmosomal components are relevant for providing a sufficient positive inotropic response to digitoxin treatment, we applied an ex vivo heart perfusion set up [2] to measure cardiac function of a previously described PG-deficient mouse model (PG KO). These animals reflect the phenotype of AC with development of arrhythmia accompanied by fibrosis, cardiac dilation and cardiac hypertrophy [24, 35]. Furthermore, it was shown that loss of PG is accompanied by a drastic reduction of DSG2 at the ICD, whereas other ICD components were not altered [35]. After establishing a baseline, the perfused hearts of PG KO mice and their wild-type (WT) littermates were treated with 100 nM and subsequently 1 µM digitoxin for 15 and 10 min, respectively, and pulse pressure and heart rate were simultaneously recorded (Fig. 1). Compared to time point 0 without treatment, the pulse pressure of WT hearts was significantly increased by perfusion with 100 nM for 15 min and 1 µM digitoxin for additional 10 min, confirming a positive inotropic response (Fig. 1a, b). In contrast, PG KO hearts failed to enhance the pulse pressure amplitude under these conditions. However, neither in WT nor in PG KO hearts the heart rate was altered by digitoxin treatment (Fig. 1c, d). To evaluate the ultrastructure of the ICDs under these conditions, we performed transmission electron microscopy (TEM) with cardiac slices of WT and PG KO mice treated with 1 µM digitoxin. Interestingly, the positive inotropic effect correlated with thickening of the ICD plaque in WT mice after digitoxin treatment, whereas this effect was abolished in PG-deficient cardiac tissue (Fig. 1e, f). In contrast, the intercellular space at the ICD and length of cell–cell junctions configured as area composita [10] was not altered in all conditions (Fig. 1g, h). Together, this suggests that treatment with the inotropic agent digitoxin modulates the structure of the ICD and that a positive inotropic response is dependent on the presence of the desmosomal molecule PG.

### Digitoxin enhances the binding force of DSG2 interactions

**Fig. 2:**
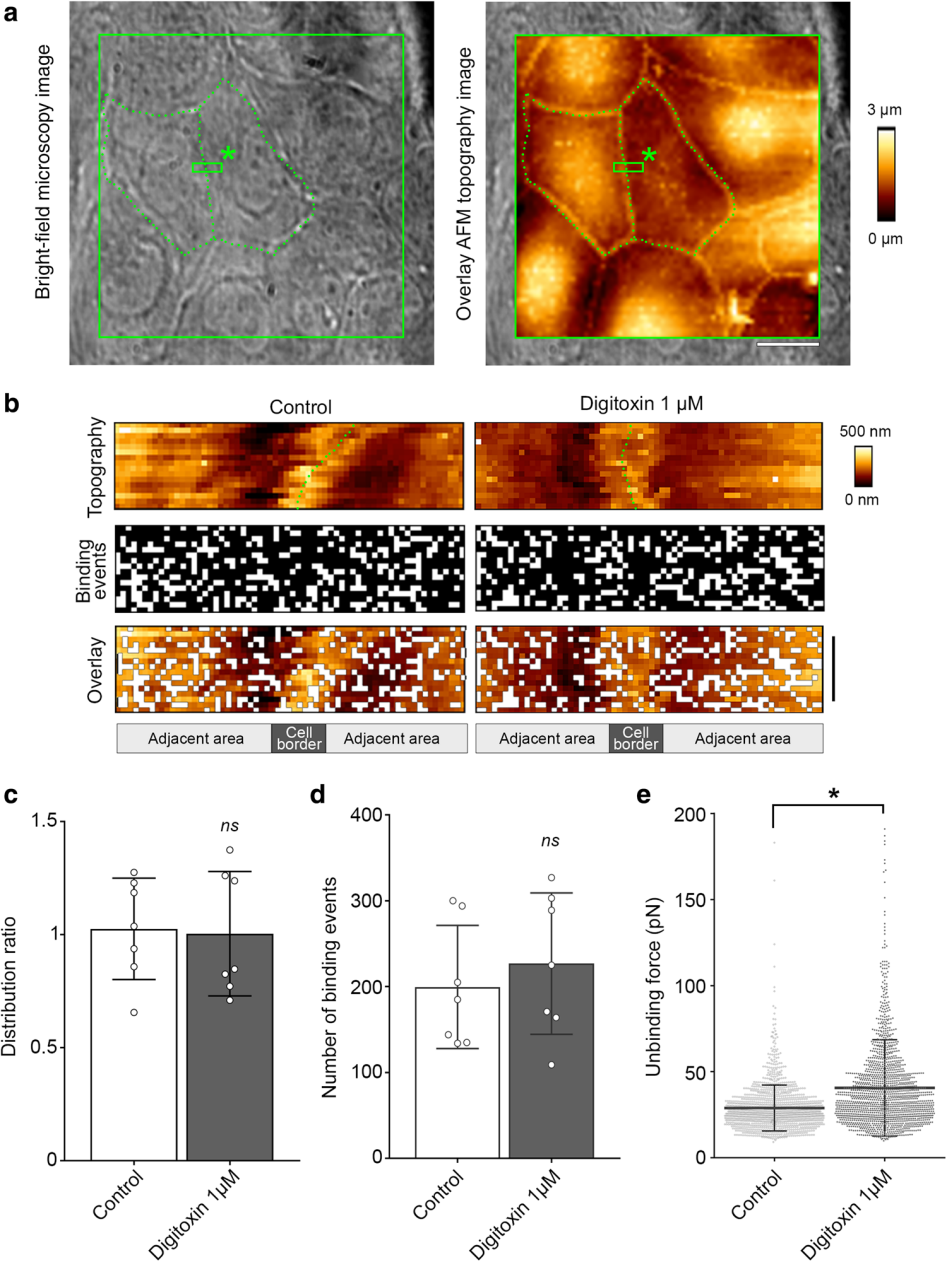
Digitoxin increases DSG2 binding force. AFM force measurements were performed with a DSG2-coated tip on living HL-1 cardiac myocytes at areas of cell–cell contact as shown in **a**, green rectangle marked with an asterisk. These scanning regions were selected from AFM topography images (example in right panel) generated on confluent cell layers as confirmed by bright-field microscopy (example in left panel). Green dotted lines exemplarily highlight boundaries of two cells in both images. Scale bar: 10 µm. Force measurements were performed before and after incubation with digitoxin 1 µM for 30–90 min. *N* = 7 independent experiments and coatings. Bars indicate mean values ± SD. **b** Representative AFM topography and binding maps depicting the distribution of DSG2-mediated binding events along the cell–cell border of HL-1 cells. Each white pixel indicates one binding event, scale bar: 1 µm. Green dotted line highlights cell–cell border. **c** Corresponding distribution ratio of DSG2 binding events is defined as binding frequency at the cell border versus the adjacent cell area. To calculate the distribution ratio, a 10-pixel-wide region at cell–cell contacts was defined as cell border as indicated in **b**. Every dot indicates one independent experiment. Two-tailed, paired Student’s *t* test, (ns)*P* > 0.05 vs. control. **d** Corresponding mean number of DSG2 binding events per force map. Every dot indicates one independent experiment. Two-tailed, paired Student’s *t* test, (ns)*P* > 0.05 vs. control. **e** Corresponding scatter blot of binding forces of all binding events. Every dot indicates the force value of one binding event. Mann-Whitney test, *P < 0.05

To evaluate whether the effect of digitoxin on contractility is paralleled with a regulation of desmosomal adhesion, the binding properties of the desmosomal transmembrane adhesion molecule DSG2 at the cell–cell contact area of living murine HL-1 cardiac myocytes (Fig. 2a) was investigated by atomic force microscopy (AFM) [35]. Briefly, a flexible cantilever with a DSG2-functionalized tip repeatedly approached the cell surface to enable an interaction of the tip-linked DSG2 with the cell surface molecules. A binding event and the force necessary to rupture this binding was subsequently determined by measurement of the cantilever’s deflection. Previous studies identified these surface molecules at least in part to be DSG2 molecules providing homophilic interactions [33, 35]. As shown for different cell types, desmosomal cadherins present on the cell surface can co-localize to desmosomal plaque proteins and intermediate filaments [43, 46, 47], which might function as a reserve pool for desmosome assembly [16, 35]). For AFM studies, these desmosomal precursors can serve as surrogates to study desmosomal adhesion. For measurements, areas containing the cell–cell contact region of adjacent HL-1 cells were selected. These regions were visible in the topography image, which was confirmed by bright-field microscopy (green dotted lines, Fig. 2a). For both, basal condition and digitoxin treatment, a homogenous distribution of DSG2 binding events was observed at the cell–cell contact area (Fig. 2b). This was further analyzed as ratio of the binding frequency directly at the cell contact area and the adjacent cell area (Fig. 2c). The total number of binding events remained unchanged (Fig. 2d). However, after addition of digitoxin, the mean force necessary to rupture the binding between the tip-coupled DSG2 and the molecules on the surface of HL-1 cells was significantly increased from 28.9 to 40.6 pN (Fig. 2e) suggesting that digitoxin enhanced the interaction force of DSG2 molecules and modified desmosome adhesion.

### Digitoxin increases cardiac myocyte cohesion

**Fig. 3:**
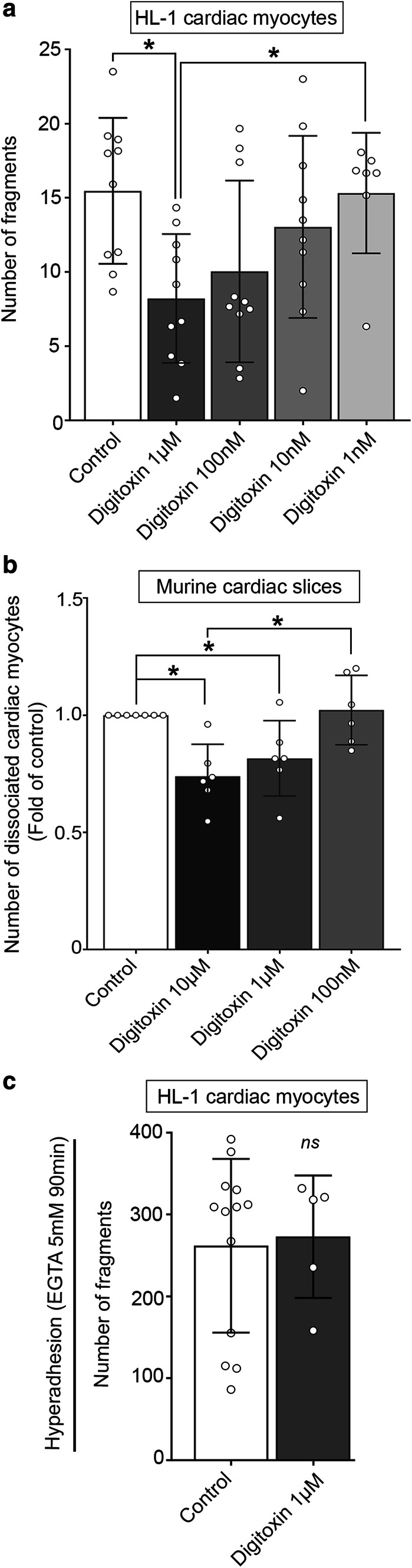
Digitoxin strengthens cardiac myocyte cohesion. Cell–cell adhesion was determined by dissociation assays measuring the degree of fragmentation of a HL-1 monolayer (**a**) or the number of intact cardiac myocytes dissociated from murine cardiac slices (**b**) in response to mechanical stress. Digitoxin was applied in the indicated concentrations for 60 min. Bars indicate mean values ± SD. Every dot represents the mean value of two to four dependent replicates. *P < 0.05. **a** *N* = 9 (control, digitoxin 1 µM, 100 nM, and 10 nM) or 7 (digitoxin 1 nM) independent experiments, respectively. One-way ANOVA with Sidak’s post-hoc test. **b** *N* = 6 independent experiments. Kruskal–Wallis with Dunn’s post-hoc test. **c** Modified dissociation assay to detect Ca^2+^-independent cell cohesion. HL-1 cells were treated with digitoxin 1 µM for 60 min with subsequent switch to low Ca^2+^ medium containing 5 mM EGTA and digitoxin 1 µM for 90 min. *N* = 5 independent experiments. Every dot represents the mean value of two to three dependent replicates. Bars indicate mean value ± SD. Mann–Whitney test, (ns)*P* > 0.05 vs. control

To further investigate the impact of digitoxin on cardiac cell–cell adhesion, we performed a dissociation assay in vitro using HL-1 cardiac myocytes and ex vivo in murine cardiac slices as previously established [35]. In keratinocytes, the dissociation assay reflects the situation in vivo in which the epidermis is subjected to mechanical stress [39]. However, it is not possible to directly translate the level of applied stress to the amount of mechanical load in vivo. The same holds true for studies in cardiac myocytes. Nevertheless, the assay is powerful since it allows to directly quantify cohesion. Digitoxin was applied to both models for 60 min in concentrations ranging from 10 µM to 1 nM (Fig. 3). In vitro as well as ex vivo, digitoxin induced a concentration-dependent increase in cell–cell adhesion, detectable as reduced fragmentation of a detached cell monolayer or decreased number of cardiac myocytes dissociated from a transversal murine heart slice after subjection to mechanical stress such as shaking or pipetting (Fig. 3a, b). In HL-1 cardiac myocytes treated with 1 µM digitoxin, number of fragments were significantly lower compared to control and 1 nM concentration. In cardiac tissue, digitoxin significantly reduced number of cells dissociated by mechanical stress at concentrations of 10 µM and 1 µM compared to control and 10 µM compared to 100 nM. This demonstrates an increase in cardiac cohesion in response to digitoxin in a cell line as well as in cardiac tissue, the latter of which exhibits correct cardiac architecture forming ICDs.

Ca^2+^-independent cadherin binding, referred to as “hyperadhesion”, was shown to be important for regulation of desmosomal adhesion during wound healing and differentiation in keratinocytes [13, 18]. Furthermore, it was observed in cardiac myocytes in response to adrenergic stimulation [35]. To evaluate if changes in Ca^2+^ dependency of adhesion are induced by digitoxin treatment, a previously established dissociation protocol [13, 35] was applied. Subsequent to the incubation with digitoxin, 5 mM of the Ca^2+^ chelator EGTA was applied to the floating HL-1 monolayer to disrupt Ca^2+^-dependent cohesion. However, after treatment with 1 µM digitoxin, no changes in the number of monolayer fragments was detectable indicating that digitoxin does not induce a hyperadhesive state of cell cohesion (Fig. 3c).

### Digitoxin induces accumulation of desmosomal molecules at cell–cell contacts

**Fig. 4:**
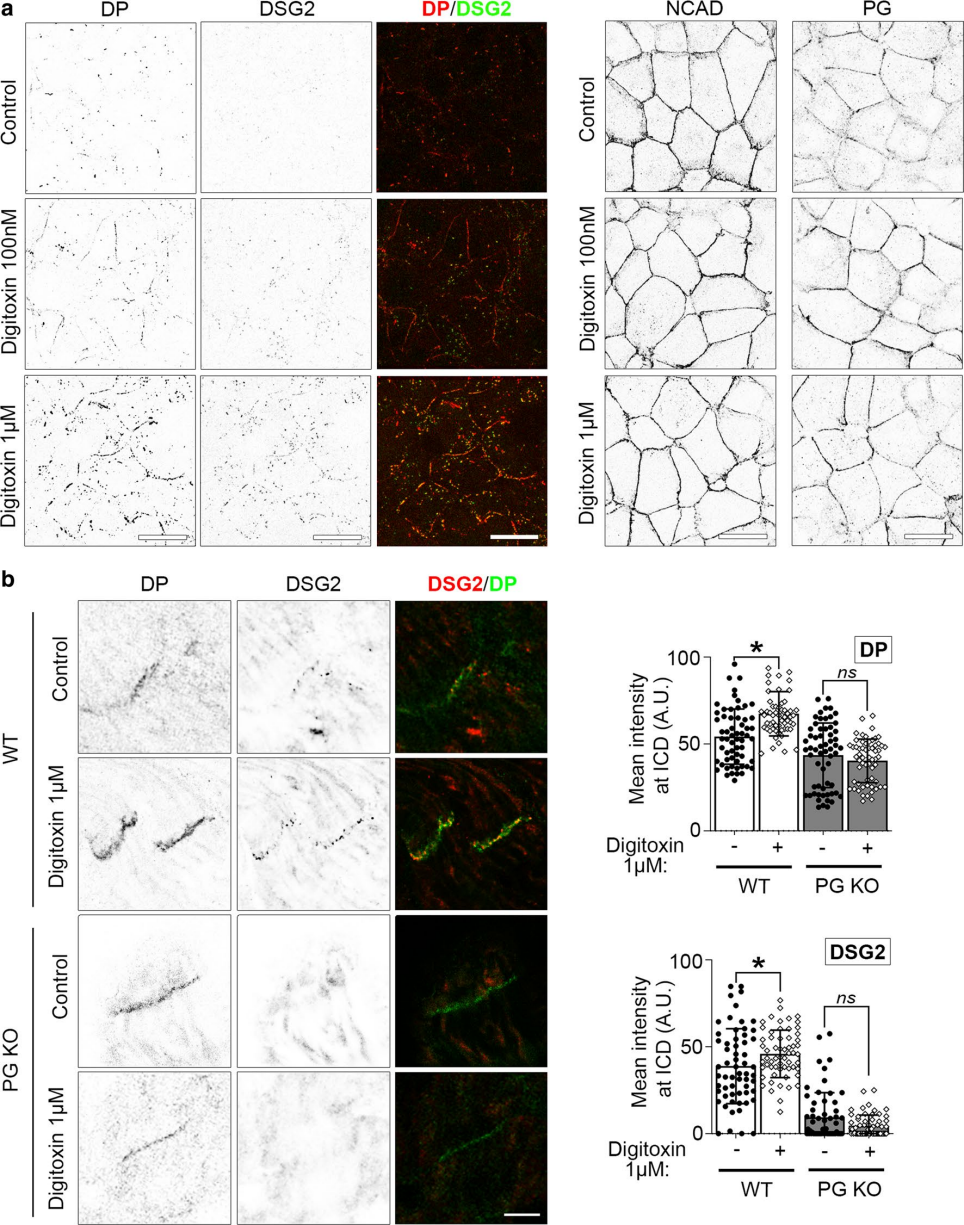
Digitoxin causes accumulation of DSG2, DP and PG at the cell–cell contacts. **a** Representative immunostaining images of HL-1 cardiac myocytes treated with digitoxin at indicated concentrations for 60 min and stained for DP (in merge: red), DSG2 (in merge: green), PG, or NCAD, scale bar: 20 µm. For better visibility, single channel images were inverted. *N* = 6 independent experiments with two dependent replicates per experiment. **b** Representative immunostaining images of ICDs of murine cardiac slices treated with digitoxin 1 µM for 60 min and stained for DP (in merge: green), DSG2 (in merge: red), scale bar 5 µm. For better visibility, single channel images were inverted. *N* = 3 mice per condition. Analysis of signal intensity of DP or DSG2 at the ICD. Every dot corresponds to one ICD, 20 ICDs per mouse measured, mean ± SD. Two-way ANOVA with Sidak’s post-hoc test. *P < 0.05, (ns)*P* > 0.05

As the presented data indicate a regulation of the adhesive function of the ICD by digitoxin, immunostaining of the desmosomal proteins DSG2, DP and PG and the adherens junction-specific adhesion molecule NCAD was performed in HL-1 cardiac myocytes and murine cardiac slices to investigate potential changes in the localization of these proteins paralleled to increased cohesion. Under baseline conditions in HL-1 cells, DSG2 and DP staining was detectable in sporadic dots along the cell–cell junction area. After digitoxin treatment, both molecules appeared to be more accumulated at cell–cell interface with elongation of signals along the cell–cell contact area (Fig. 4a). This effect was concentration-dependent with a more pronounced accumulation upon treatment with 1 µM digitoxin compared to 100 nM. PG staining appeared partially continuous with diffuse regions at  the cell–cell contact area under control conditions, whereas digitoxin treatment increased continuous PG staining at cell–cell contacts. In contrast, the mostly continuous staining of NCAD at the cell–cell interfaces showed no alteration after digitoxin treatment.

Similar to effects found in HL-1 cells, 1 µM digitoxin induced accumulation of DSG2 and DP staining at the ICD of WT mouse hearts (Fig. 4b). In PG KO heart slices, DSG2 was drastically reduced at ICDs under baseline conditions as published before [35] and was further not altered by digitoxin treatment. In addition, accumulation of DP, which was present at the ICD at baseline conditions in both genotypes, was abolished in response to digitoxin in PG KO compared to WT cardiac slices. As myofilaments are essential for cardiac contractility and are inserting in the ICD plaque, morphology of the desmin intermediate and actin filament system was investigated (Supplementary Fig. 1). Tissue deficient for PG compared to WT displayed no alteration in morphology for both filaments neither under baseline conditions as described before [35], nor after treatment with digitoxin. However, morphological changes beyond immunostaining resolution or functional alterations cannot be ruled out by these data.

**Fig. 5:**
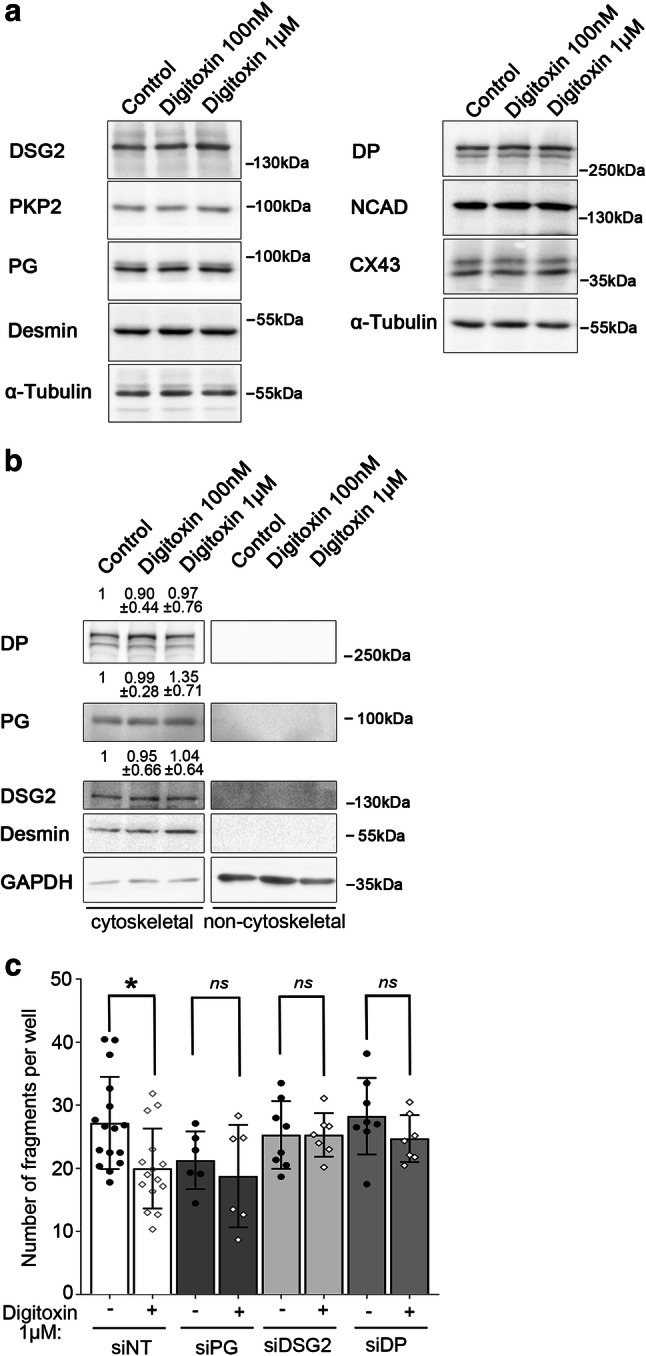
Effect of digitoxin is dependent on desmosomal proteins. **a** Representative Western blot analysis of indicated ICD proteins in HL-1 cells treated with digitoxin at concentrations of 100 nM or 1 µM for 60 min. α-Tubulin served as loading control. *N* = 6 independent experiments. **b** Triton-X-extraction assay to separate cytoskeletal and non-cytoskeletal-bound fractions of HL-1 cardiac myocytes treated with digitoxin at concentrations of 100 nM or 1 µM for 60 min. GAPDH served for the non-cytoskeletal and desmin for the cytoskeletal-bound fraction as loading and separation control. Values above the lanes depict the mean band intensity by densitometric quantification compared to the respective loading control as fold of control ± SD, *N* = 5 independent experiments. Kruskal–Wallis with Dunn’s post-hoc test. *P < 0.05, (ns)*P* > 0.05. **c** Dissociation assay of HL-1 cells transfected with non-targeting siRNA (siNT) or siRNAs to reduce protein levels of PG (siPG), DSG2 (siDSG2), or DP (siDP) and treated with digitoxin 1 µM for 60 min. Bars indicate mean value ± SD. Every dot represents the mean value of two to three dependent replicates. Two-way ANOVA with Sidak’s post-hoc test, *P < 0.05. *N* = 15 (siNT), 7 (siDSG2, siDP) or 6 (siPG) independent experiments, respectively

Additional Western blot analysis revealed that the overall expression levels of DSG2, DP, PG and other ICD proteins (PKP2, desmin, NCAD, CX43) were not altered in response to treatment with digitoxin in HL-1 cells (Fig. 5a). Further, biochemical separation of a cytoskeletal from a non-cytoskeletal-bound protein fraction by triton-X-extraction assay was performed to test for alterations in anchorage of desmosomal components. However, this assay revealed no difference in the distribution of DSG2, DP and PG between both fractions (Fig. 5b) with all three proteins mainly present cytoskeletal bound.

Together, these data demonstrate that treatment with digitoxin induces an accumulation especially of desmosomal proteins at cell–cell junctions, whereas the expression levels and cytoskeletal anchorage of desmosomal but also other ICD components seem to remain unchanged. This indicates an accumulation of desmosomal components at the cell–cell interface more by shifting or clustering of existing molecules than changes in the amount of proteins.

### Increase of cardiac myocyte cohesion by digitoxin is dependent on the presence of desmosomal proteins

To reveal if the increase in cell–cell adhesion induced by digitoxin treatment is directly linked to desmosomal molecules, the protein expression of DSG2, DP and PG was suppressed by specific small interfering RNAs (siRNAs). Compared to control conditions, silencing of each of the three desmosomal proteins abrogated the reduction of monolayer fragmentation and thus the increase of cell cohesion in response to digitoxin treatment (Fig. 5c). Knock-down efficiency was confirmed by Western blot analysis for every experiment (Supplementary Fig. 2). Interestingly, silencing of DP or PG decreased the expression levels of DSG2, which is in line with DSG2 to be important for the effect of digitoxin on cardiac cohesion.

Because phosphorylation of PG was shown to modulate desmosomal adhesion in cardiac myocytes [35] as well as in other tissue [54], we investigated the phosphorylation state of PG in response to digitoxin using the Phos-Tag approach. In this SDS-PAGE-based approach, the phosphorylated protein fraction migrates slower compared to non- or lower-phosphorylated proteins. Changes in the phosphorylation state of proteins are depicted as alterations in band intensity or migration height. Under basal conditions, PG was separated in two fractions, indicating at least one phosphorylation site of PG. After treatment with digitoxin at 100 nM or 1 µM, no changes in the phosphorylation state of PG were observed (Supplementary Fig. 3A). Similarly, DP was evaluated under the same conditions and no alterations were detected (Supplementary Fig. 3B). In summary, these data show that the increase of cell–cell adhesion by digitoxin is dependent on desmosomal proteins, whereas phosphorylation of PG or DP seems not to be involved.

### Increase of cardiac myocyte cohesion by digitoxin is dependent on ERK1/2 signaling

**Fig. 6:**
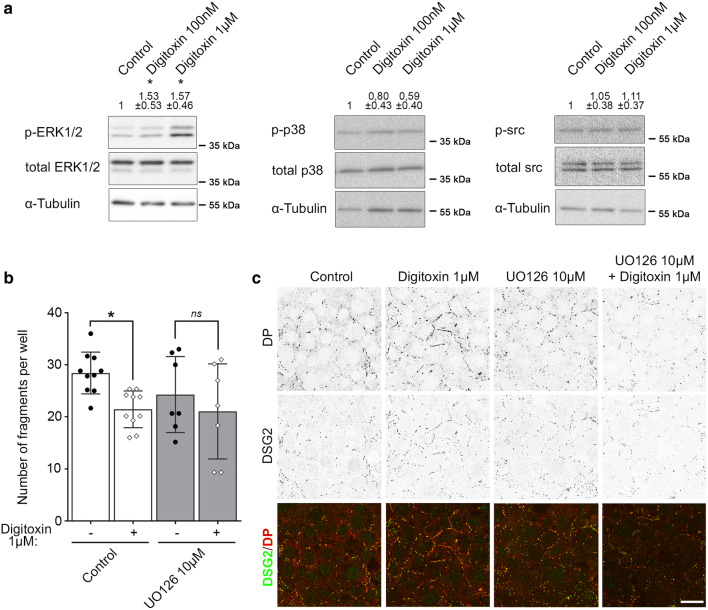
Effect of digitoxin is dependent on phosphorylation of ERK1/2. **a** Western blot analysis of HL-1 cells treated with digitoxin 100 nM or 1 µM for 60 min to reveal phosphorylation state of ERK1/2, p38MAPK (p38) and Src. α-Tubulin served as loading control. Values above the lanes depict the mean band intensity by densitometric quantification compared to the respective total protein as fold of control ± SD, *N* = 6 independent experiments. Kruskal–Wallis with Dunn’s post-hoc test. *P < 0.05. **b** Dissociation assay of HL-1 cells pre-treated with 10 µM UO 126 for 60 min to inhibit ERK1/2 phosphorylation with subsequent application of 1 µM digitoxin for 60 min. Bars indicate mean value ± SD. Every dot represents the mean value of two to four dependent replicates. Two-way ANOVA with Sidak’s post-hoc test, *P < 0.05, (ns)*P* > 0.05. *N* = 9 (control) or 6 (UO 126 10 µM) independent experiments, respectively. **c** Representative immunostaining images of DSG2 (in merge: green) and DP (in merge: red) in HL-1 cardiac myocytes pre-treated with UO 126 10 µM for 60 min with subsequent application of digitoxin 1 µM for 60 min; scale bar: 20 µm. For better visibility, single channel images were inverted. *N* = 6 independent experiments with two dependent replicates per experiment

To identify the underlying mechanism by which positive inotropy induced by digitoxin causes a reorganization of desmosomal proteins and increased cell–cell adhesion, we biochemically analyzed the modulation of kinases known to be involved in the regulation of desmosomal adhesion [31, 35, 49]. HL-1 cells were treated with digitoxin 100 nM or 1 µM for 60 min and the phosphorylation state of ERK1/2, p38MAPK and tyrosine kinase Src was assessed (Fig. 6a). This analysis revealed increased levels of phosphorylated ERK1 as well as ERK2 after treatment with both digitoxin concentrations. In contrast, analysis of p38MAPK and Src revealed no alteration in kinase phosphorylation in response to digitoxin treatment. To verify the relevance of ERK1/2 signaling for the digitoxin effect described in this study, UO126 was used as MEK1/2 inhibitor to block ERK1/2 activation [8]. Applying UO126 to HL-1 cardiac myocytes in a concentration range from 1 to 50 µM for 60 min, digitoxin-induced ERK1/2 phosphorylation was reduced at 1 µM UO126 and completely inhibited by co-treatment with 10 µM UO126 or higher concentrations (Supplementary Fig. 4). In the dissociation assay, pre-incubation with UO126 for 60 min was efficient to block the digitoxin-induced increase in cell cohesion (Fig. 6b). Furthermore, the digitoxin-dependent accumulation of DSG2 and DP at the cell–cell contact area was abrogated by UO126 treatment (Fig. 6c).

In addition to the mentioned pathways, PKC signaling was shown to modulate desmosomal assembly and function [12, 23, 40]. As the active form of PKC associates with the plasma membrane [20], we biochemically separated the membrane from the cytosolic fraction and probed for the conventional PCKα and the novel PKCε isoform (Supplementary Fig. 5A). For both isoforms, no changes in fractioning were detectable after incubation with digitoxin, indicating no PKC activation by this treatment.

As further digitoxin was described to regulate Ca^2+^ levels, which serves as an important signaling molecule in various signaling pathways, we evaluated Ca^2+^ levels in HL-1 cardiac myocytes in response to digitoxin treatment. However, applying the FURA-2 sensor [25], no alterations in FURA-2 (340/380 nm) ratio were detectable after application of 1 µM and 10 µM digitoxin with regard to baseline levels and the amplitude of Ca^2+^increase during contraction (Supplementary Fig. 5B). Additional treatment with caffeine served as positive control.

Taken together, these data demonstrate that digitoxin leads to increased cell cohesion and reorganization of desmosomal proteins via activation of the ERK1/2 signaling pathway, whereas other pathways tested seem not to be involved.

### Adrenergic signaling induces phosphorylation of PG and ERK1/2, while digitoxin only activates ERK1/2 signaling

Adrenergic stimulation is one of the main regulators of cardiac function and physiological condition regulating heart rate, contractility and excitability. Its downstream signaling via cAMP was shown to increase ICD cohesion with accumulation of DSG2, DP and PG at the ICD via phosphorylation of PG at S665 [35]. This pathway seems to be important for adaption of cell–cell cohesion to increased mechanical load by enhanced contractility. Given the positive inotropic effect of digitoxin and the data on cardiac myocyte cohesion presented in this study, we aimed to evaluate the interplay of both signals on the mechanical coupling of ICDs.

**Fig. 7:**
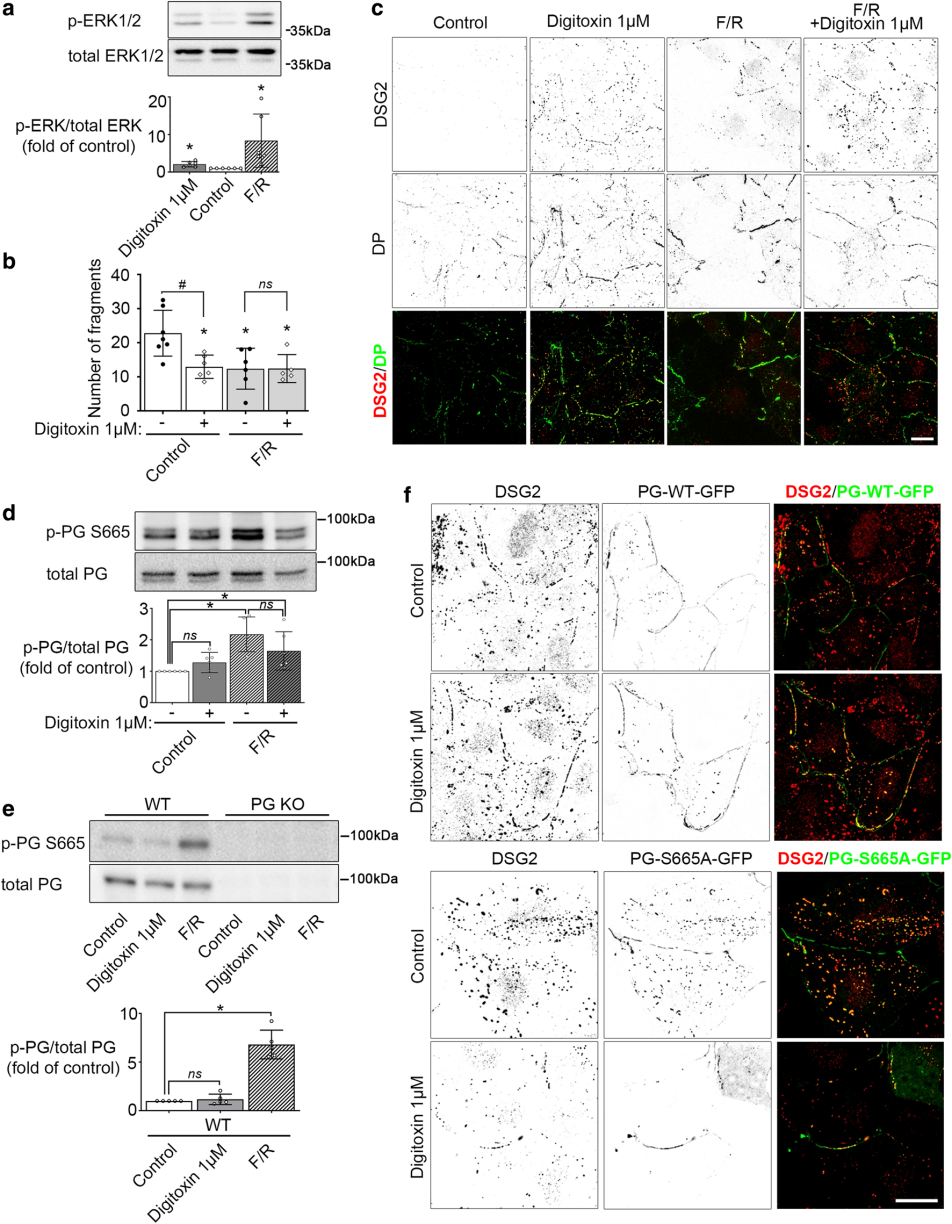
Digitoxin and adrenergic signaling induces ERK1/2 activation without additive effects. **a** Western blot analysis of HL-1 cells treated with digitoxin 1 µM or *F*/*R* 5 µM/10 µM for 60 min to reveal phosphorylation state of ERK1/2. Bar graphs depict the mean band intensity by densitometric quantification compared to the respective loading control as fold of control ± SD, *N* = 6 independent experiments. Kruskal–Wallis with Dunn’s post-hoc test. *P < 0.05 vs. control. **b** Dissociation assay of HL-1 cells treated with digitoxin 1 µM, FR 5 µM/10 µM or combination of both for 60 min. Bars indicate mean value ± SD. Every dot represents the mean value of two to three dependent replicates. Two-way ANOVA with Tukey’s post-hoc test, *P < 0.05 vs control, ^#^*P* < 0.05, (ns)*P* > 0.05. *N* = 7 (control), 6 (digitoxin 1 µM) or 5 (*F*/*R*, *F*/*R* + digitoxin 1 µM) independent experiments, respectively. **c** Representative immunostaining images of DSG2 (in merge: red) and DP (in merge: green) in HL-1 cardiac myocytes treated with digitoxin 1 µM, *F*/*R* 5 µM/10 µM or combination of both for 60 min. Scale bar: 10 µm. For better visibility, single channel images were inverted. *N* = 4 independent experiments with two dependent replicates per experiment. Western blot analysis of HL-1 cells (**d**) or murine cardiac slices (**e**) treated with digitoxin 1 µM or *F*/*R* 5 µM/ 10 µM for 60 min to reveal phosphorylation state of PG at S665. Bar graphs depict the mean band intensity by densitometric quantification compared to the total protein as fold of control ± SD, *N* = 6 (control, digitoxin 1 µM, *F*/*R* + digitoxin 1 µM) or 5 (*F*/*R*) independent experiments, respectively in **d**. *N* = 5 mouse hearts per genotype in **e**. Kruskal–Wallis with Dunn’s post-hoc test. *P < 0.05, (ns)*P* > 0.05. **f** Representative immunostaining images of DSG2 (in merge: red) in HL-1 cardiac myocytes overexpressing GFP-tagged (in merge: green) PG phospho-deficient at S665 (PG-S665A-GFP) or wild-type PG (PG-WT-GFP) as control. Cells were treated with digitoxin 1 µM for 60 min. Scale bar: 10 µm. For better visibility, single channel images were inverted. *N* = 3 independent experiments with two dependent replicates per experiment

First, activation level of ERK1/2 in response to adrenergic signaling was evaluated. To activate the adrenergic pathway, a combination of the adenylyl cyclase activator forskolin and phosphodiesterase inhibitor rolipram was applied for 60 min to increase levels of the second messenger cAMP. Western blot analysis revealed significantly increased phosphorylation of ERK1/2 by *F*/*R* (Fig. 7a). To investigate if this activation of ERK1/2 by both stimuli can be additive on the functional level, dissociation assays and immunostaining were performed. As described before, digitoxin as well as *F*/*R* treatment significantly reduced fragmentation of HL-1 cell monolayer with accumulation of DSG2 and DP at the cell contact area (Fig. 7b, c). However, combination of both agents did not induce an additional reduction of fragments or enhanced recruitment of DSG2 or DP towards the cell–cell contacts.

Phosphorylation of PG at S665 is one key step for the regulation of ICD cohesion by adrenergic stimulation. However, Phos-Tag data revealed no phosphorylation of PG in response to digitoxin (Supplementary Fig. 3). To investigate this in more detail, a monoclonal mouse antibody raised against a phospho-peptide containing the sequence of PG-S665 was generated. Western blot analysis applying this phosphosite-specific antibody confirmed phosphorylation of PG at S665 by *F*/*R* in HL-1 cardiac myocytes and in cardiac slices, whereas digitoxin had no effect on PG phosphorylation when applied alone or in combination with *F*/*R* (Fig. 7d, e). Probing PG KO samples confirmed PG specificity of the generated antibody. In contrast to adrenergic stimulation [35], accumulation of DSG2 at the cell contact area was not blocked in cells overexpressing the phospho-deficient fusion protein PG-S665A-eGFP (Fig. 7f) in response to digitoxin treatment, which is in line with a pathway independent from phosphorylation of PG.

In summary, both digitoxin and adrenergic signaling induce activation of ERK1/2, whereas digitoxin did not trigger phosphorylation of PG at S665. This suggests two parallel pathways efficient to regulate mechanical coupling of the ICD.

## Discussion

**Fig. 8:**
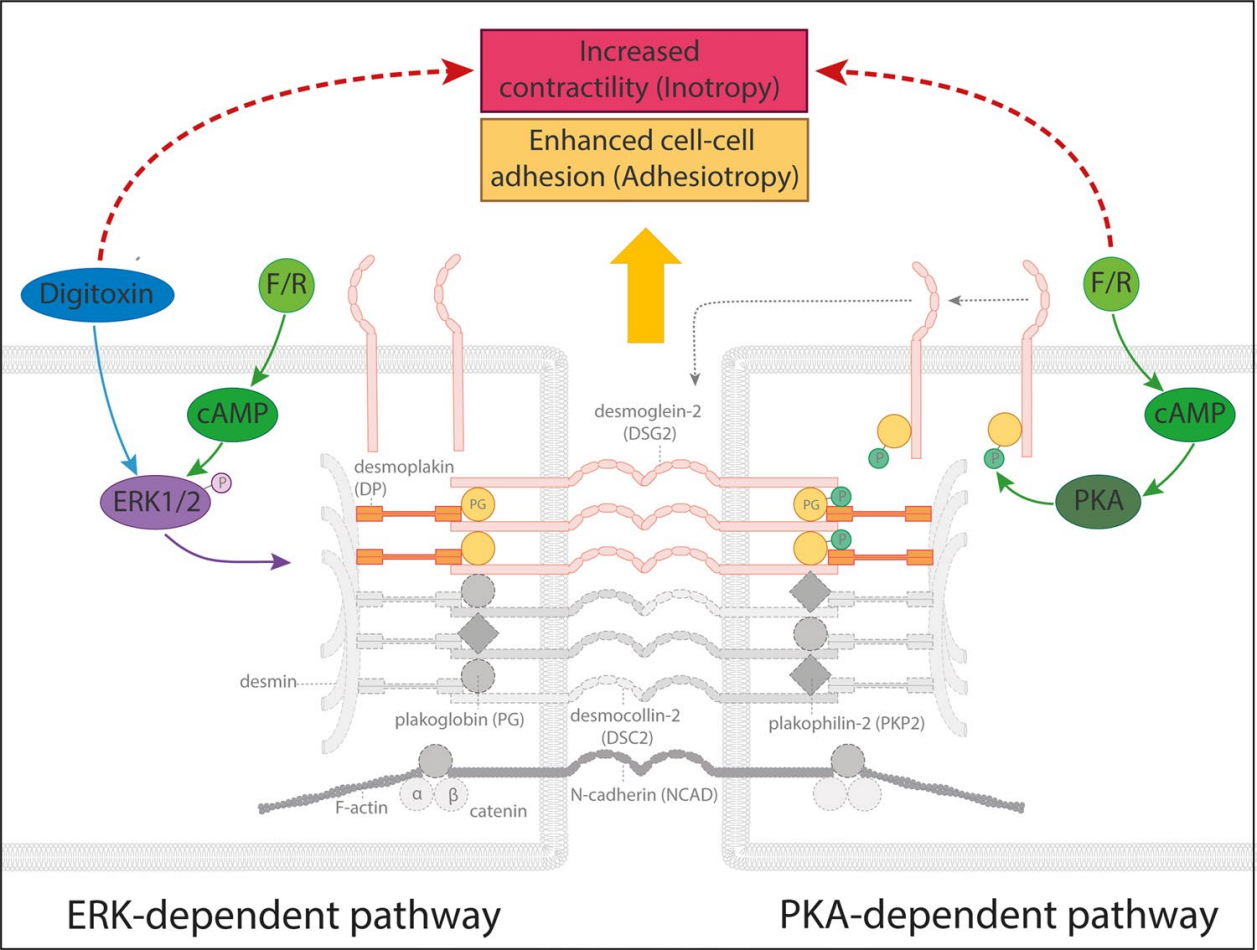
Schematic of the effects of digitoxin and adrenergic signaling on ICD proteins. Schematic summary of the effect of digitoxin (blue) or adrenergic signaling via *F*/*R* (green) on desmosomal components of the ICD. Both positive inotropic pathways induce a recruitment of desmosomal proteins with strengthening of the mechanical coupling of the ICD either via ERK1/2 activation (left side) or via PKA-dependent phosphorylation of PG with shift of desmosomal precursors from the cell surface towards the cell–cell interface (right side). Baseline situation is depicted in grey with dashed contour. Alterations by the two pathways (ERK- or PKA-dependent) depicted in color with continuous contour. Encircled “P” indicates phosphorylation of the respective protein

In this study, we demonstrate that the positive inotropic agent digitoxin increases cardiac myocyte cohesion with enhanced DSG2 binding force and accumulation of the desmosomal proteins DSG2, DP and PG at the cell–cell contact area paralleled with ultrastructural thickening of the ICD plaque. The described effect was dependent on the presence of these desmosomal proteins. In comparison to adrenergic signaling, which strengthens cell–cell adhesion via a PKA-dependent phosphorylation of PG and additionally induces phosphorylation of ERK1/2, the effect of digitoxin on cohesion was solely dependent on ERK1/2 activation. Because digitoxin failed to increase pulse pressure in PG-deficient mouse hearts, these data indicate that intact cardiac cohesion is required to enable sufficient positive inotropic response to digitoxin treatment (Fig. 8).

Previous studies provided evidence that enhanced cardiac myocyte cohesion is a novel function of adrenergic signaling in the heart, referred to as positive adhesiotropy [35]. Here, adrenergic stimulation via PKA-dependent phosphorylation of PG at S665 induced a rapid shift of the desmosomal proteins DSG2, PG, and DP presumably from a reserve pool at the cell surface area where no direct contact to a neighboring cell is present towards the cell–cell interface to strengthen mechanical coupling of the ICD. This mechanism seemed to be important to adapt cohesion of the ICD to the increased mechanical load by the paralleled positive inotropic effect of adrenergic stimulation. In this study, we show vice versa that a positive inotropic agent is effective to induce enhanced ICD cohesion. Analyzing the underlying mechanism, we found that enhanced cardiac cohesion by digitoxin is dependent on expression of desmosomal proteins with DSG2 playing a central role. Furthermore, an increased strength of DSG2 binding events after digitoxin treatment was revealed by AFM as also described for adrenergic stimulation. This was accompanied by a recruitment of DSG2 as well as DP and PG towards the cell–cell junctions and thickening of the ICD plaque. As no alterations in protein amount or cytoskeletal anchorage of these proteins were detectable, this points to changes in localization of existing proteins with accumulation and clustering at the cell–cell interface to increase cell cohesion.

Focusing in this study on the desmosomal components of the ICD, the signaling pathway identified here might also have implications on recently discovered ICD proteins with adhesive function such as the β1 subunit of cardiac sodium channel [44]. This can be of interest as this protein is involved in ephaptic electrical coupling and action potential propagation between cardiac myocytes. However, this need to be addressed by further studies.

Interestingly, when the distribution of DSG2 binding events within the cell contact area was resolved using AFM force mapping, no differences were found within 2.5 µm from the cell border. This is different to the effects induced by adrenergic signaling where additionally a redistribution of DSG2 binding events from the cell surface to the cell–cell interface was present [35]. This indicates that digitoxin, similar to adrenergic stimulation, triggered increased DSG2 binding strength but in contrast to adrenergic signaling did not enhance binding through redistribution of DSG2 from the cell surface towards the junctions. Even though the mechanisms of how DSG2-mediated binding can be enhanced on the cell surface are not clear, processes which also happen during desmosome assembly such as protein cluster formation, cytoskeletal anchorage or conformational changes, e.g., by posttranslational modifications, are conceivable based on published data [35, 46, 47].

Previously, we have shown that adrenergic signaling increased cell cohesion at least in part via direct PKA-mediated phosphorylation of PG [35]. Investigating the signaling pathways relevant for the increase of desmosomal cohesion in response to digitoxin, ERK1/2 appears to be critical, since ERK1/2 was activated by digitoxin and upstream MEK1/2 inhibition abrogated digitoxin-induced effects on cell cohesion and desmosome component reorganization. In contrast, no alterations in the activation state of Src, p38MAPK or PKC were detectable. Even though no alterations in Ca^2+^ levels by digitoxin treatment were detectable via FURA-2, localized accumulation of Ca^2+^ not detectable by this approach are possible [3, 37] and might induce Ca^2+^-dependent signaling and downstream effects. However, no activation of the Ca^2+^-dependent kinase PKCα was found in this study. It is known that digitoxin and other cardiac glycosides act via inhibition of Na^+^/K^+^-ATPase [38] and are efficient to activate the corresponding signalosome including the Ras-Raf-MEK1/2-ERK1/2 cascade [26, 42]. There is also evidence that ERK1/2 can be activated by digitoxin through the PI3K pathway [52, 56]. On the other hand, ERK1/2 is known to be regulated in a manner dependent on desmosomal function. Thus, loss of a desmosomal protein such as DSG2 or DP induced phosphorylation of ERK1/2 in cardiac myocytes [21] or in a pancreatic cancer cell line [19]. In addition, activation of ERK1/2 is also involved in the pathogenesis of pemphigus, an autoimmune blistering skin disease caused by autoantibodies against DSG1 and DSG3 [48]. Together with recent data showing that phosphorylation of ERK1/2 was triggered in a desmosome-dependent manner in response to mechanical stress [22], this points to desmosomes as mechano-sensing structures, initiating downstream signaling pathways including ERK1/2. Taken in account that ERK1/2 inhibition abrogated the elevation of cell–cell adhesion by digitoxin, ERK1/2 signaling downstream of the Na^+^/K^+^-ATPase signalosome might directly enhance desmosomal function. Vice versa, disturbed desmosomal adhesion, e.g. by increased mechanical load via elevated contractility might trigger activation of ERK1/2 to modulate desmosomal adhesion.

In line with this concept, adrenergic signaling was shown to evoke ERK1/2 activation in addition to the described phosphorylation of PG at S665. In contrast, digitoxin did not induce phosphorylation of PG and was efficient to accumulate DSG2 at the cell contacts, despite expression of a PG mutant phospho-deficient at S665. However, combination of adrenergic signaling and digitoxin revealed no enhanced effects compared to single components. These observations indicate that two different mechanisms exist to enhance cell cohesion with adrenergic signaling triggering both pathways, whereas digitoxin activates ERK1/2 signaling only. However, targets of ERK1/2 signaling regarding desmosomes are not clear, yet. Direct phosphorylation of desmosomal proteins by ERK1/2 is possible as respective phosphorylation motifs can be found in various components including PG, DP or PKP2 [5]. In addition, indirect effects on desmosomes via regulation of associated proteins, e.g., from the group of cytoskeletal proteins, or activation of downstream kinases such as FAK can be speculated [30, 55].

The data presented highlight the role of desmosomal components for quick adaption of cardiac cell–cell adhesion in response to increased contraction force. Thus, strengthened ICD cohesion is triggered by agents enhancing pulse pressure of the heart in parallel. As indicated by data in perfused hearts of PG KO mice in this and a previous study [35], the increase in cohesion may be essential for a positive inotropic effect of adrenergic stimulation as well as after treatment with cardiac glycosides. Thus, positive adhesiotropy or at least intact desmosomal adhesion seems to be a requirement for sufficient positive inotropy and heart function. Focusing on cardiac glycosides and modulators of adrenergic signaling as therapeutics in congestive heart failure [28], the positive adhesiotropic mechanism might be important for therapeutic out-come [41]. Thus, if strengthening of cohesion in parallel to enhanced contractility is abrogated by defective desmosomes, e.g., via mutations as present in AC, the cohesion of the ICD might not be adapted to the increased mechanical load. Potentially, this may enhance detachment of ICDs as shown in [35], with subsequent cardiac myocyte cell death and fibrosis. Moreover, physical exercise may aggravate mechanical uncoupling of cardiac myocytes and thereby trigger malignant ventricular arrhythmias and progression of the disease [4]. This points to a contraindication of positive inotropic agents in cardiac pathologies with an adhesive defect such as in AC and is in line with β-adrenergic blockers as first line therapy. On the other hand, a recent study indicated direct stabilization of desmosomal adhesion via a linking peptide to be a promising treatment option for AC [33].

Taken together, the data provided here demonstrate enhanced cardiac myocyte cohesion as a new effect of the positive inotropic agent digitoxin on heart function. Moreover, it is evident that intact desmosomal adhesion is required for digitoxin to induce a positive inotropic effect and possibly the capability of digitoxin to enhance pulse pressure may be dependent on its positive effect on cardiac myocyte cohesion. Thus, the study provides further insight in the mode of action of cardiac glycosides and the role of positive adhesiotropy for cardiac function.

## Electronic supplementary material

Below is the link to the electronic supplementary material.

Supplementary file1 (PDF 867 kb)

Supplementary file2 (PDF 471 kb)
