# Supplementary material for: The inotropic agent digitoxin strengthens desmosomal adhesion in cardiac myocytes in an ERK1/2-dependent manner
Source: Basic Res Cardiol. 2020 Jun 17;115(4):46. doi: 10.1007/s00395-020-0805-3 (PMC7299919; doi:10.1007/s00395-020-0805-3)

SUPPLEMENTARY DATA- Basic Research in Cardiology

Uncropped Western blots

The inotropic agent digitoxin strengthens desmosomal adhesion in cardiac myocytes in an ERK1/2-dependent manner

Camilla Schinner[1,2+], Silvana Olivares-Florez[1+], Angela Schlipp[1], Sebastian Trenz[1], Manouk Feinendegen[1], Heinrich Flaswinkel[3], Ellen Kempf[1], Desalegn Tadesse Egu[1], Sunil Yeruva[1], and Jens Waschke[1*]

[1]Faculty of Medicine, Ludwig-Maximilians-Universität (LMU) Munich, Munich, Germany

[2]Department of Biomedicine, University of Basel, Basel, Switzerland

[3]Department of Biology II, Ludwig-Maximilians-Universität (LMU) Munich, Munich, Germany

[+]Authors contributed equally.

[*]Corresponding author:

Jens Waschke, Faculty of Medicine, Ludwig-Maximilians-Universität (LMU) Munich, Pettenkofferstraße 11, 80336 Munich, Germany; Phone: +49-89-2180-72610; Fax: +49-89-2180-72602; jens.waschke@med.uni-muenchen.de

Uncropped Western blots with marker images corresponding to bands shown in the indicated figures

Band signal image shown on the left, overlay of bands with corresponding marker image shown on the right. To enable more protein incubations some blots were cut at 70 kDa and incubated separately. Bands appearing from previous antibody incubations are indicated in brackets and smaller font size. Blots are shown in order of antibody incubation. Antibody species: “m” = mouse; “rb” = rabbit. Prestained protein ladder PageRuler plus (10 – 250 kDa, Thermo Scientific) was used as size marker for all blots.

Supplementary Data – uncropped Western blots

Figure 5A

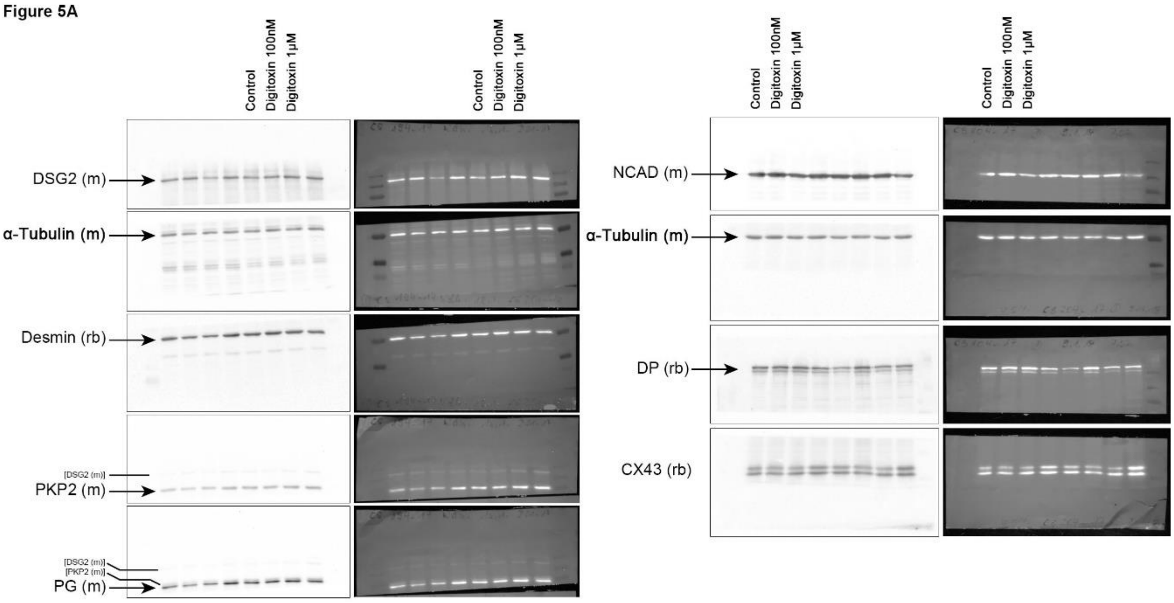

Figure 5B

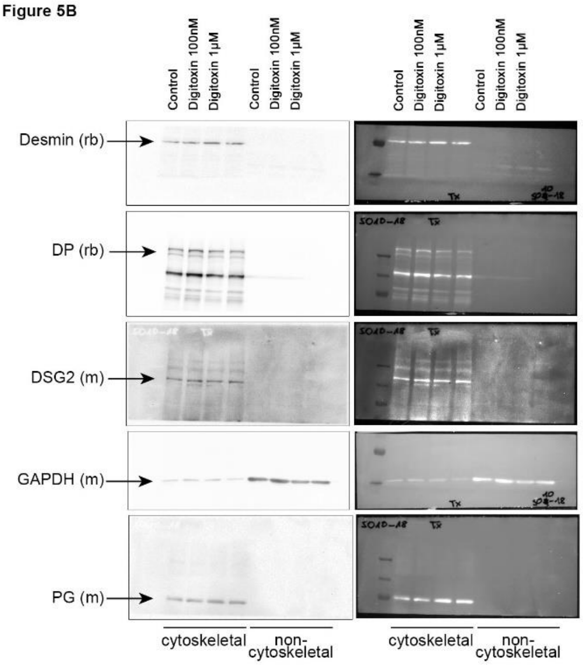

Figure 6A

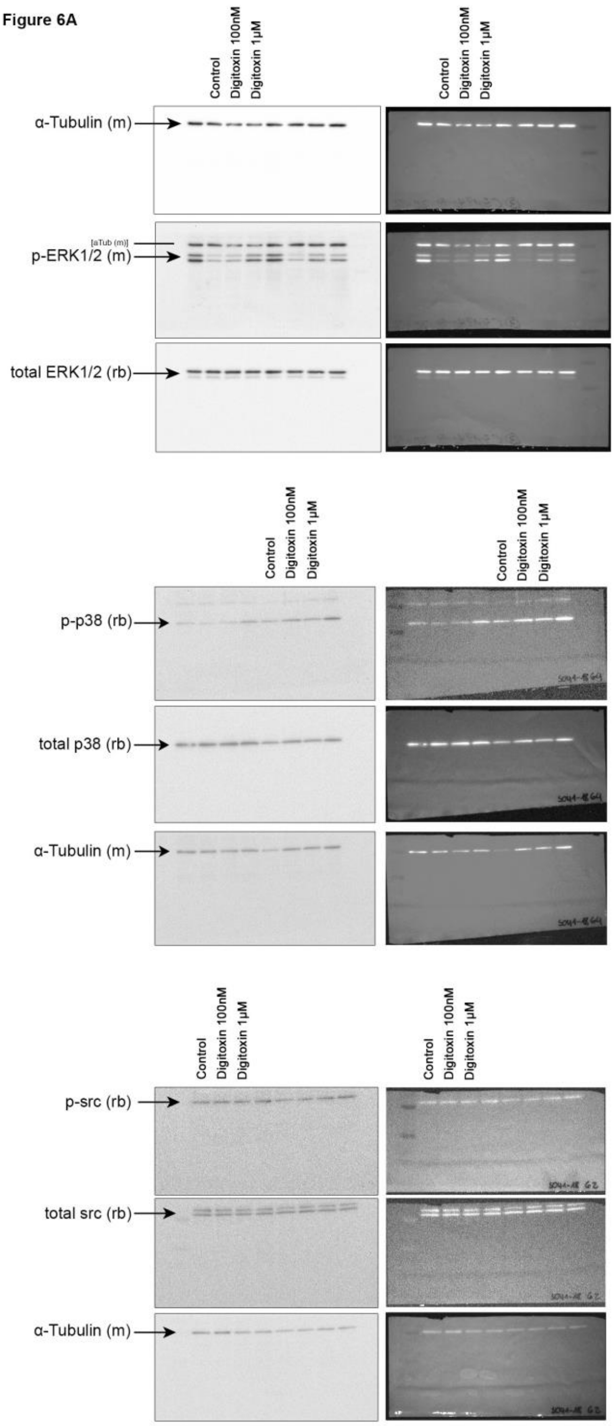
Control
Digitoxin 100nM
Digitoxin 1µM
α-Tubulin (m)
[αTub (m)]
p-ERK1/2 (m)
total ERK1/2 (rb)
p-p38 (rb)
total p38 (rb)
α-Tubulin (m)
p-src (rb)
total src (rb)
α-Tubulin (m)

Figure 7A

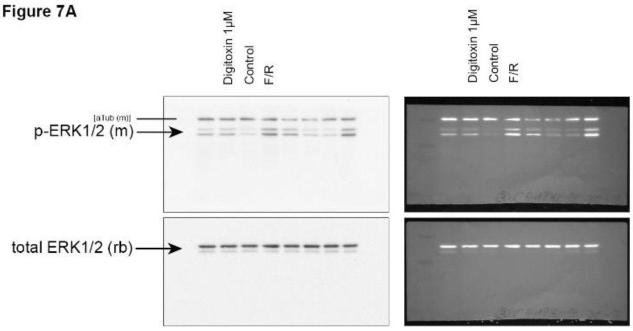

Figure 7D

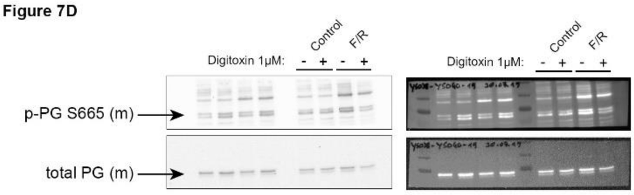

Figure 7E

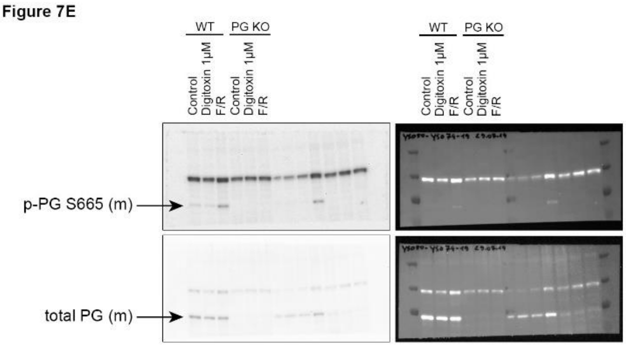

Supplementary Data – uncropped Western blots

Supplementary Figure 2

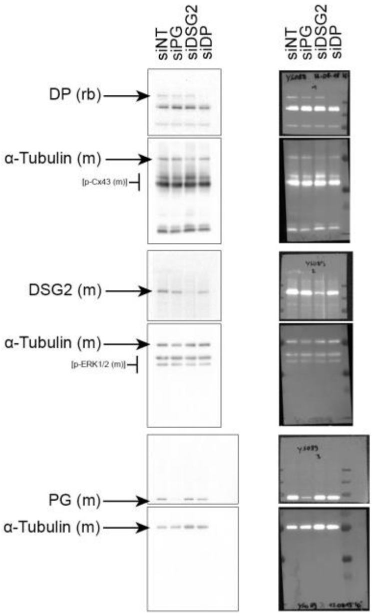

Supplementary Data – uncropped Western blots

Supplementary Figure 3A

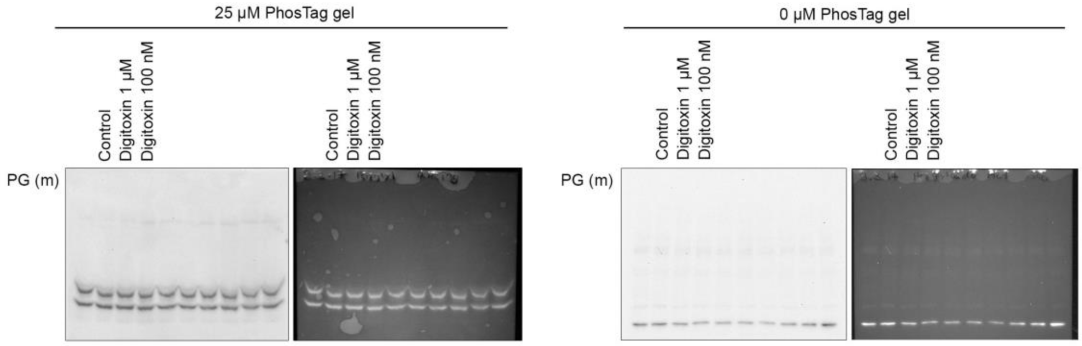

Supplementary Figure 3B

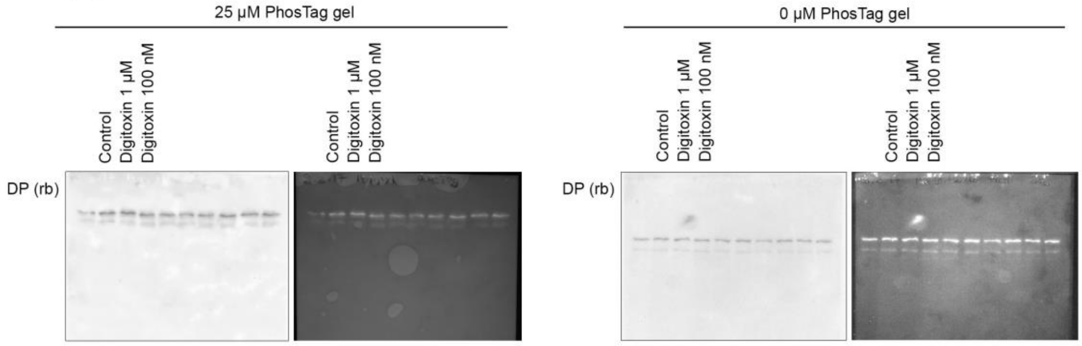

Supplementary Data – uncropped Western blots

Supplementary Figure 4

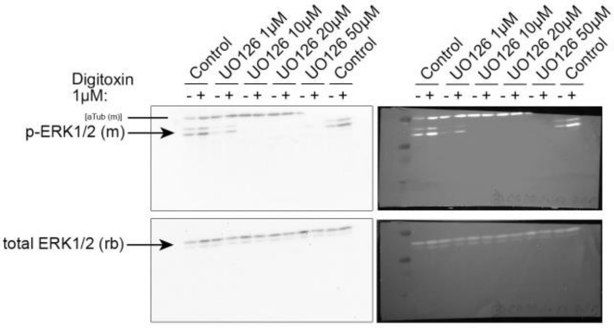

Supplementary Data – uncropped Western blots

Supplementary Figure 5A

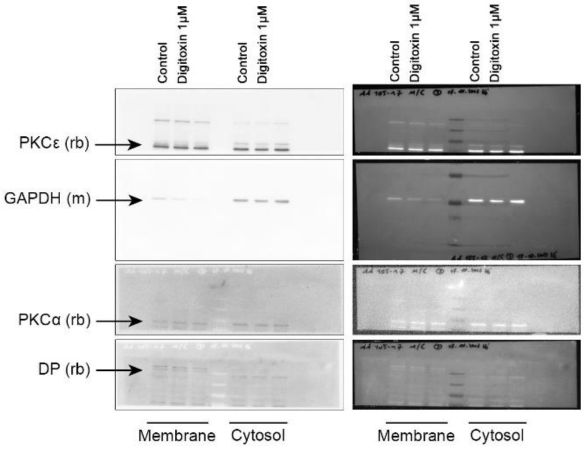

Supplement: Supplementary file 1 — Supplementary file1 (PDF 867 kb) [file 395_2020_805_MOESM1_ESM.pdf]
