# Supplementary material for: The inotropic agent digitoxin strengthens desmosomal adhesion in cardiac myocytes in an ERK1/2-dependent manner
Source: Basic Res Cardiol. 2020 Jun 17;115(4):46. doi: 10.1007/s00395-020-0805-3 (PMC7299919; doi:10.1007/s00395-020-0805-3)
Supplement: Supplementary file 2 — Supplementary file2 (PDF 471 kb) [file 395_2020_805_MOESM2_ESM.pdf]

SUPPLEMENTARY DATA- Basic Research in Cardiology

The inotropic agent digitoxin strengthens desmosomal adhesion in cardiac myocytes in an ERK1/2-dependent manner

Camilla Schinner[1,2+], Silvana Olivares-Florez[1+], Angela Schlipp[1], Sebastian Trenz[1], Manouk Feinendegen[1], Heinrich Flaswinkel[3], Ellen Kempf[1], Desalegn Tadesse Egu[1], Sunil Yeruva[1], and Jens Waschke[1*]

[1]Faculty of Medicine, Ludwig-Maximilians-Universität (LMU) Munich, Munich, Germany

[2]Department of Biomedicine, University of Basel, Basel, Switzerland

[3]Department of Biology II, Ludwig-Maximilians-Universität (LMU) Munich, Munich, Germany

[+]Authors contributed equally.

[*]Corresponding author:

Jens Waschke, Faculty of Medicine, Ludwig-Maximilians-Universität (LMU) Munich, Pettenkofferstraße 11, 80336 Munich, Germany; Phone: +49-89-2180-72610; Fax: +49-89-2180-72602; jens.waschke@med.uni-muenchen.de

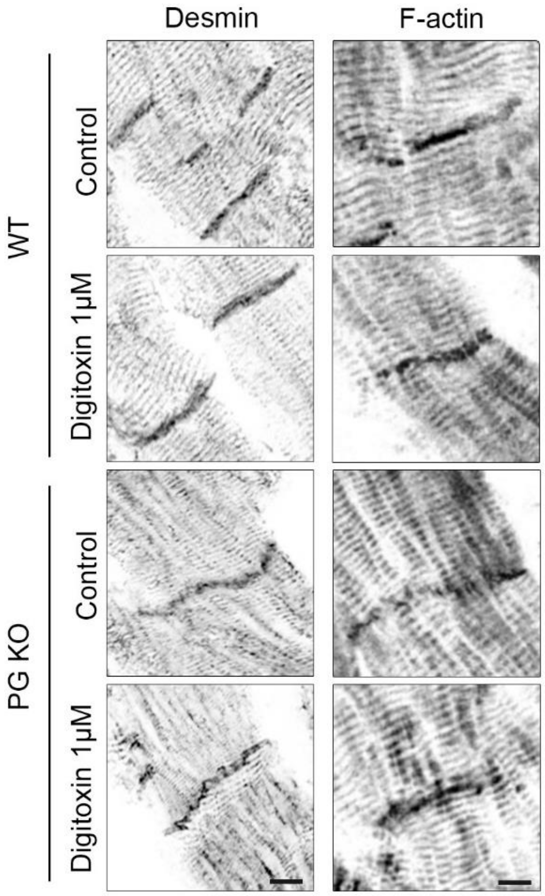

Supplementary Figure 1. Digitoxin does not alter morphology of myofilaments. Representative immunostaining images of ICDs of murine cardiac slices treated with digitoxin 1 µM for 60 min and stained for desmin or f-actin, scale bar 5 µm. For better visibility, single channel images were inverted. N = 3 mice per condition.

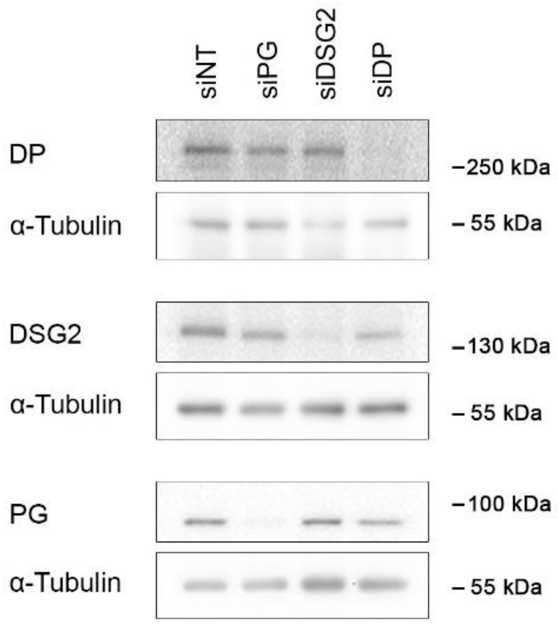

Supplementary Figure 2. Reduced levels of PG, DSG2 and DP by siRNA.

Representative Western blots corresponding to experiments in Figure 5c to confirm reduced protein levels of indicated proteins. α-Tubulin served as loading control.

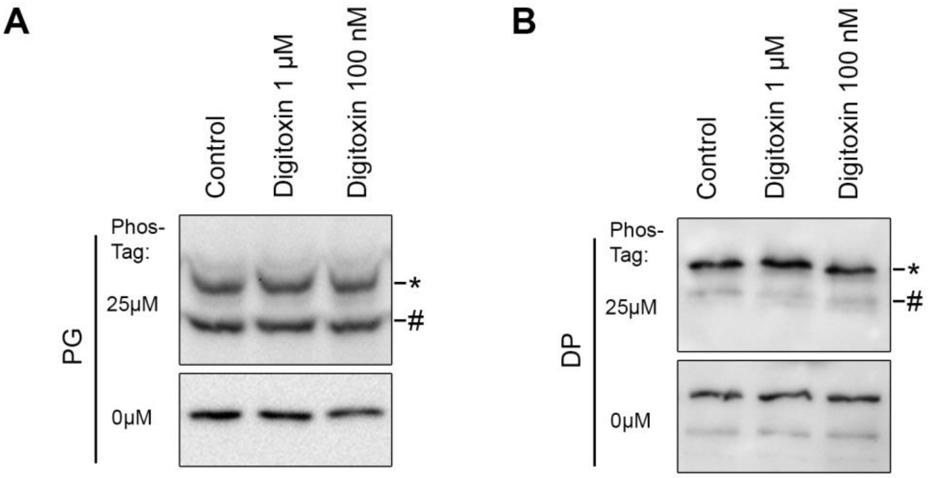

Supplementary Figure 3. Digitoxin has no effect on the phosphorylation state of PG or DP. Separation of phosphorylated proteins by Phos-Tag in HL-1 cells treated with digitoxin at concentrations of 100 nM or 1 µM for 60 min. Phosphorylation of PG (**A**) or DP (**B**) is visible in the 25 µM Phos-Tag as higher migrating bands (*) vs. the lower non- or lower-phosphorylated migrating bands (#). Gels without addition of Phos-Tag reagent served as control to exclude protein fragmentation. N = 6 independent experiments.

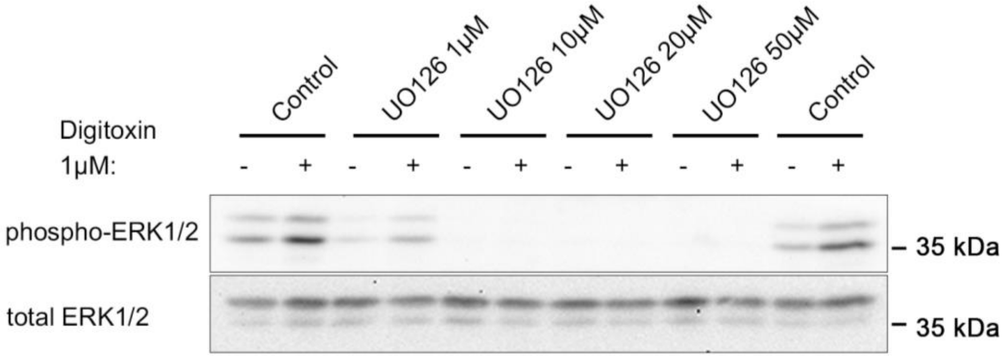

Supplementary Figure 4. UO 126 inhibits ERK1/2 phosphorylation in a concentration-dependent manner. Representative Western blot analysis to reveal phosphorylation state of ERK1/2 in HL-1 cardiac myocytes pre-treated with the MEK1/2 inhibitor UO 126 at indicated concentrations for 60 min with subsequent application of digitoxin 1 µM for 60 min. N = 6 independent experiments.

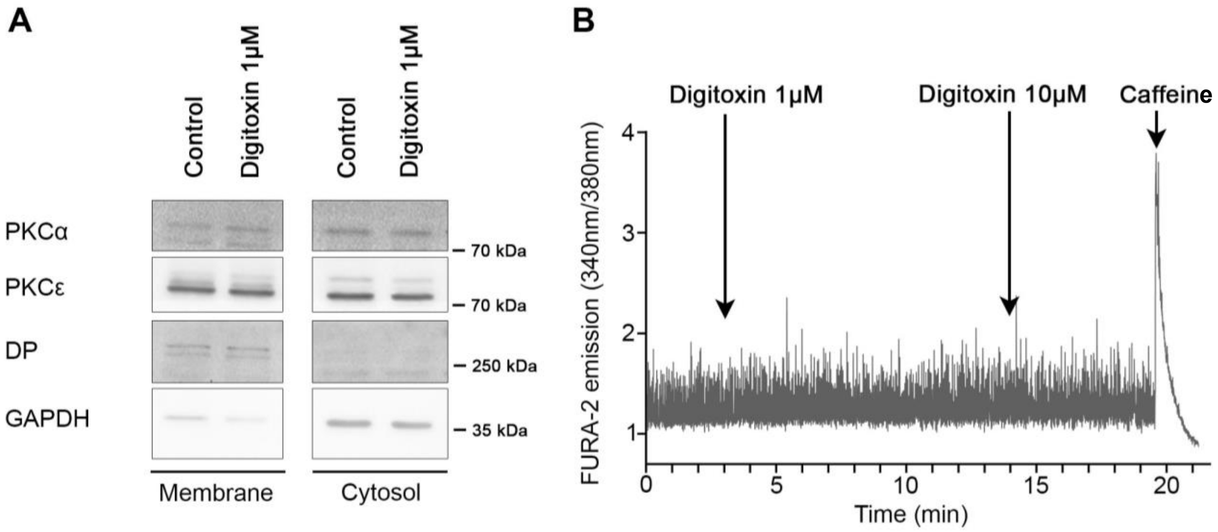

Supplementary Figure 5. Digitoxin did not alter Ca^{2+} levels or PKC activation. (**A**) Representative membrane isolation to reveal activation state of PKCα and PKCε in HL-1 cardiac myocytes treated with digitoxin 1 µM for 60 min. GAPDH served for the cytosolic and DP for the membrane fraction as loading and separation control. N = 3 independent experiments. (**B**) Representative FURA-2 measurements (ratio 340nm/380nm) to determine Ca^{2+} levels in HL-1 cardiac myocytes treated with digitoxin 1 µM and 10 µM as indicated. Caffeine 10 µM served as positive control. N = 7 independent experiments.

Supplementary Methods

Immunostaining

To visualize protein distribution, confluent HL-1 cells were treated as indicated and fixed in 2 % paraformaldehyde (PFA) in PBS for 10 min, permeabilized with 0.1 % Triton-X-100 in PBS for 5 min, and blocked with 3 % bovine serum albumin / 10 % normal goat serum (Sigma-Aldrich) in PBS for 60 min. Immunostaining of murine cardiac slices was performed as previously described [1]. Briefly, after incubation with corresponding reagents, cardiac tissue was snap frozen in liquid nitrogen and cut with a cryostat (Cryostar NX70, Thermo Fisher Scientific) in 7 µm thin sections. Probes were then heated to 37°C for 8 min, washed in PBS and fixed in 2 % PFA for 10 min. Then slices were permeabilized with 1 % Triton-X-100 for 60 min, washed in PBS and blocked with 3 % bovine serum albumin/10 % normal goat serum in PBS for 60 minutes. The following primary antibodies were incubated at 4 °C over night: polyclonal rabbit anti-DSG2 (Progen Biotechnik, Heidelberg, Germany, #610121), monoclonal mouse anti-PG (Progen, #61005), monoclonal mouse anti-DP1/2 (Progen, #61003), polyclonal rabbit anti-desmin (Abcam, #ab32362) and monoclonal mouse anti-NCAD (BD Transduction, #610921). Cy2-, and Cy3- conjugated goat anti-rabbit or goat anti-mouse secondary antibodies (Dianova, Hamburg, Germany) were incubated for 60 min at room temperature. F-actin was stained with phalloidin-Alexa Fluor 488 (Thermo Fisher Scientific, #A12379) for 60 min at room temperature. 1.5 % n-propylgallate/ 60 % glycerol (Sigma-Aldrich) in PBS was used as mounting medium. Samples were imaged and analyzed with the Leica SP5 confocal microscope equipped with a 63x oil objective using LAS-AF software (all Leica microsystems). A maximum intensity projection of 10 consecutive images with 0.3 µm step height is shown for HL-1 cells.

Western blot analysis

HL-1 cells were treated as indicated and washed in cold PBS and lysed in ice-cold SDS-lysis buffer (25 mM 4-(2-hydroxyethyl)piperazine-1-ethanesulfonic acid (HEPES), 2 mM

ethylenediaminetetraacetic acid (EDTA), 25 mM sodium fluoride (NaF), 1 % sodiumdodecylsulfate (SDS), pH 7.4) supplemented with a protease-inhibitor cocktail (Complete-O, Roche) and PhosSTOP (Roche). Protein concentration was determined by BCA protein assay kit (Pierce, Thermo Scientific, Waltham, Germany) according to manufacturer´s protocol. 10 or 20 µg protein were denaturized in Laemmli buffer at 95 °C for 5 min and loaded on a 10 % SDS-polyacrylamide gel. After electrophoresis, proteins were transferred to a nitrocellulose membrane (Novex, Thermo Fisher Scientific) and blocked in 5 % non-fat milk in tris buffered saline, with TBS-Tween 20 buffer. The primary antibodies were diluted in 5 % non-fat milk or bovine serum albumin in TBS-T buffer and incubated at 4°C over night. The following primary antibodies were used: polyclonal rabbit anti-ERK1/2 (Cell signaling, Danvers, USA, #9102), polyclonal rabbit anti-phospho-ERK1/2 (Cell signaling, #4370), polyclonal rabbit anti-p38MAPKAP (Cell signaling, #9212), polyclonal rabbit anti-phospho-p38MAPK (Cell signaling, #9211), polyclonal rabbit anti-phospho-src (Cell signaling, #2101), polyclonal rabbit anti-src (Cell signaling, #2123), monoclonal mouse anti-DSG1/2 (Progen, #61002), monoclonal mouse anti-PG (Progen, #61005), monoclonal rabbit anti-desmin (Abcam, #ab32362), polyclonal rabbit anti-DP1/2 (Santa Cruz Biotechnology Inc., Santa Cruz, USA, #sc-33555), polyclonal rabbit anti-CX43 (Sigma-Aldrich, #SAB4501175), monoclonal mouse anti-NCAD (BD Transduction, #610921), monoclonal mouse anti-PKP2 (Progen, #651167), monoclonal mouse anti-GAPDH (Aviva Systems Biology, San Diego, USA, #OAEA00006), monoclonal mouse anti-α-tubulin (Abcam, Cambridge, UK, #ab7291), monoclonal mouse anti-phosho-PG S665 (clone 1B8). Horseradish peroxidase-conjugated goat anti-rabbit and anti-mouse secondary antibodies (Dianova) were incubated at room temperature for 120 min. Proteins were visualized by the ECL-method. Densitometric band analysis was performed with Image Studio Lite Ver. 5.2 (LI-COR Biosciences, Bad Homburg, Germany).

Supplementary References

1. Schinner C, Vielmuth F, Rotzer V, Hiermaier M, Radeva MY, Co TK, Hartlieb E, Schmidt A, Imhof A, Messoudi A, Horn A, Schlipp A, Spindler V, Waschke J. Adrenergic Signaling Strengthens Cardiac Myocyte Cohesion. *Circ Res* 2017;**120**:1305-1317.
